# Bioactive Prenyl- and Terpenyl-Quinones/Hydroquinones of Marine Origin †

**DOI:** 10.3390/md16090292

**Published:** 2018-08-21

**Authors:** Pablo A. García, Ángela P. Hernández, Arturo San Feliciano, Mª Ángeles Castro

**Affiliations:** Department of Pharmaceutical Sciences, Pharmaceutical Chemistry Section, CIETUS/IBSAL, Faculty of Pharmacy, University of Salamanca, E-37007 Salamanca, Spain; pabloagg@usal.es (P.A.G.); angytahg@usal.es (A.P.H.); artsf@usal.es (A.S.F.)

**Keywords:** meroterpenoids, mixed metabolites, terpenylquinones, terpenylhydroquinones, bioactivity

## Abstract

The sea is a rich source of biological active compounds, among which terpenyl-quinones/hydroquinones constitute a family of secondary metabolites with diverse pharmacological properties. The chemical diversity and bioactivity of those isolated from marine organisms in the last 10 years are summarized in this review. Aspects related to synthetic approaches towards the preparation of improved bioactive analogues from inactive terpenoids are also outlined.

## 1. Introduction

Terpenyl-quinones/hydroquinones (TQs/THQs) constitute a family of mixed biogenesis metabolites widespread in nature, either as primary or secondary metabolites in many organisms. Compounds like vitamin K, tocopherols, ubiquinones, or plastoquinones belong to this family, and they play important roles in oxidative metabolism, such as in electron transport chain or in photosynthesis [1]. Many other TQs/THQs have been isolated from different sources, and they are often referred as meroterpenoids because of their biosynthesis through a mixed polyketide-isoprenoid pathway. A wide variety of biological activities has also been described for them, mainly cytotoxic and antioxidant properties that are been related with their involvement in redox cycling and/or Michael-1,4-addition reactions. Those quinone derivatives with a unique biosynthetic origin, either from a polyketide or isoprenoid pathway, are not considered in this revision.

Many of those metabolites were isolated from marine organisms, such as brown algae and sponges, and these have been covered by previous specific reviews, which were mainly organized according the source organism [1,2,3,4,5,6]. In this review, we focus on the structure and bioactivity of TQs/THQs of marine origin described along the last 10 years (January 2008–April 2018). They have been organized according the size and type of the terpenoid fragment, ranging from simple isoprene units to large terpenoids, and according the size and nature of the quinone moiety, with most of them being *p*-benzoquinone (BQ)/1,4-hydroquinone (HQ) derivatives. Often, they have varied carbon skeletons originating from intermolecular cyclizations and/or rearrangements of the initial terpenoid precursors, leading to unique cyclic structures. We consider aspects related not only with their structure or marine source, but also with their biological properties, and in some cases with those synthetic approaches towards the natural products and/or their bioactive analogues. It should be noted that the numbering used for these compounds is not uniform; sometimes the terpenoid part is numbered as usual and the polyketide core with primes, and other times, the quinone fragment is numbered as a continuation of the terpenoid system or even the other way around. Due to the difficulties that unifying it could cause, we decided to keep the numbering system used in the original articles, indicating in the figures those positions that are mentioned in the text.

## 2. Meroterpenoids with Simple Isoprene Units

**Figure 1:**
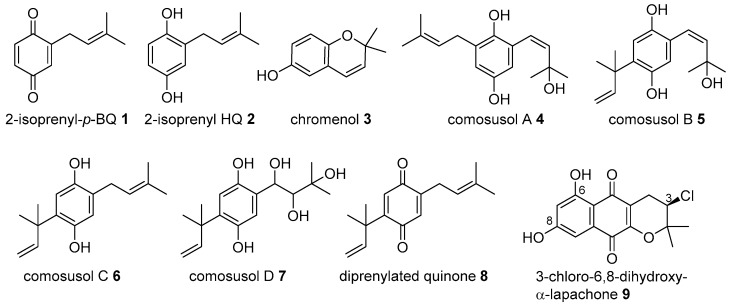
Structures of meroterpenoids with simple isoprenyl units.

Few examples of simple meroterpenoids, with only one or two isoprenyl units, have been described since the initial isolation of the isoprenylquinone **1**, HQ **2**, and the chromenol **3** (Figure 1) from the ascidian *Aplidium californicum* in the late 1970s [7]. They were covered in a previous review [3] but in addition to the previously reported cytotoxic properties [7], the anti-inflammatory activity of **1** had been described more recently, after its isolation from fresh specimens of *Aplidium* sp. collected near Leigh Harbor, New Zealand [8].

Several bis-isoprenyl HQ derivatives, comosusols A–D (**4**–**7**), and the diprenylated quinone **8** (Figure 1) were isolated from the brown algae *Sporochnus comosus* [9]. They were screened for their cytotoxic activities against the four human tumor cell lines MCF-7 (breast), SF-268 (central nervous system), H460 (lung), and HT-29 (colon), and on normal mammalian cells CHO-K1 (Chinese hamster ovary). All of them were cytotoxic in the μM range, with **5** being the most potent, with GI_50_ values between 5 and 6 μM against the panel of cell lines tested [9].

The same diprenylated quinone **8** was described by the first time from the New Zealand brown alga *Perithalia capillaris* and it was efficiently synthesized by Moody using two consecutive Claisen rearrangements that proceeded with both high chemo- and regioselectivity [10].

An example of a naphthoquinone (NQ) of marine origin with only one isoprenyl group is 3-chloro-6,8-dihydroxy-α-lapachone (**9**) (Figure 1), a new natural compound isolated from the marine-derived *Streptomyces* sp. SCSIO 10428, obtained from a sediment sample collected from Xieyang Island, China [11].

## 3. Meroterpenoids with Open-Chain and Cyclic Monoterpenoids

Meroterpenoids with a monoterpenyl unit were isolated from diverse marine organisms, either as HQ or as quinone derivatives. In addition, the original geranyl chain can be as such with different oxidation degrees, or it can be cyclized, forming carbocycles and/or chromene systems by ring-closure between one of the phenolic groups of the HQ and a double bond of the terpenoid.

**Figure 2:**
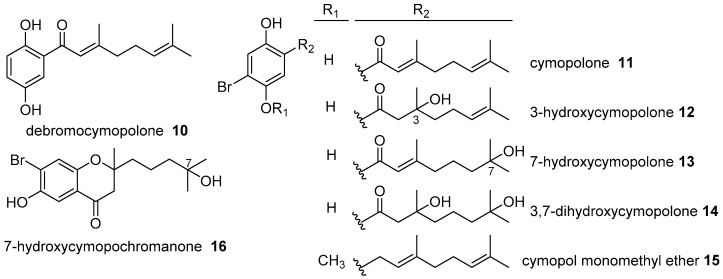
Cymopol type meroterpenoids isolated from *Cymopolia barbata*.

From the green alga *Cymopolia barbata*, collected at the northeastern coast of Jamaica, a new cymopol type metabolite that did not contain a bromine atom, debromocymopolone (**10**), was isolated together with the previously identified brominated compounds cymopolone (**11**), 3-hydroxycymopolone (**12**), 7-hydroxycymopolone (**13**), 3,7-dihydroxycymopolone (**14**), cymopol monomethyl ether (**15**) and 7-hydroxycymopochromanone (**16**) [12] (Figure 2).

**Figure 3:**
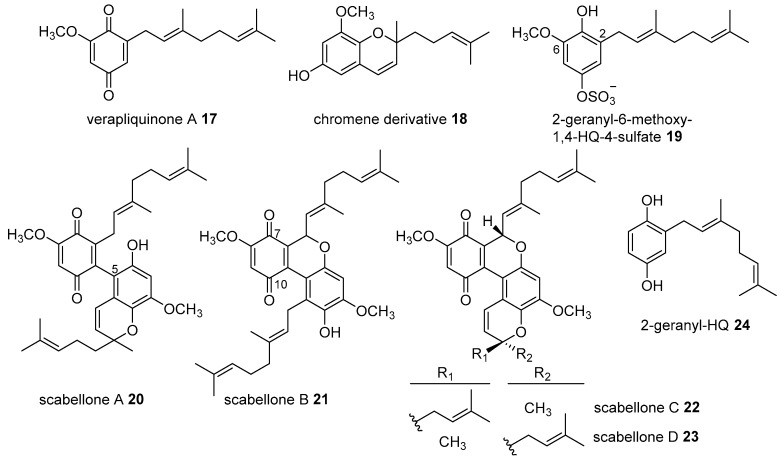
Structures of *Aplidium* meroterpenoids **17**–**24.**

Copp et al. isolated and identified several new and known BQ/HQ derivatives, with a geranyl substituent, from the ascidian *Aplidium scabellum*, collected at Rabbit Island in New Zealand [13]. Among the known metabolites were verapliquinone A (**17**) and the chromenol **18** (Figure 3), which were identified in the same extract fraction as the new compound 2-geranyl-6-methoxy-1,4-HQ-4-sulfate (**19**). The next fractions contained a family of new pseudodimeric meroterpenoids, named scabellones A–D (**20**–**23**) (Figure 3). Scabellone A (**20**) contained a 5-substituted chromenol fragment, while the others contained a benzo[*c*]chromene-7,10-dione scaffold, which was particularly rare among natural products [13]. In terms of biogenesis, it seemed reasonably that they proceeded via dimerization of 2-geranyl-6-methoxybenzoquinone and/or 2-geranyl-6-methoxyhydroquinone, and in fact, the same authors performed a biomimetic dimerization synthesis of scabellones A and B starting from 2-geranyl-6-methoxyhydroquinone with oxygen in pyridine [14].

Chromenol **18**, HQ-sulfate **19**, and scabellone B (**21**) were found to have anti-inflammatory activity mediated by inhibition of neutrophil superoxide production [13], activity that was also detected for the simple HQ **24** (Figure 3), isolated from fresh specimens of New Zealand ascidian *Aplidium* sp. [8]. Scabellone B (**21**) was also evaluated against the parasites *Trypanosoma brucei*, *Trypanosoma cruzi*, *Leishmania donovani*, and *Plasmodium falciparum*, showing certain selectivity towards *P. falciparum* with IC_50_ 4.8 μM, and low cytotoxicity on a L6 rat myoblast cell line (IC_50_ 65 μM) [13].

**Figure 4:**
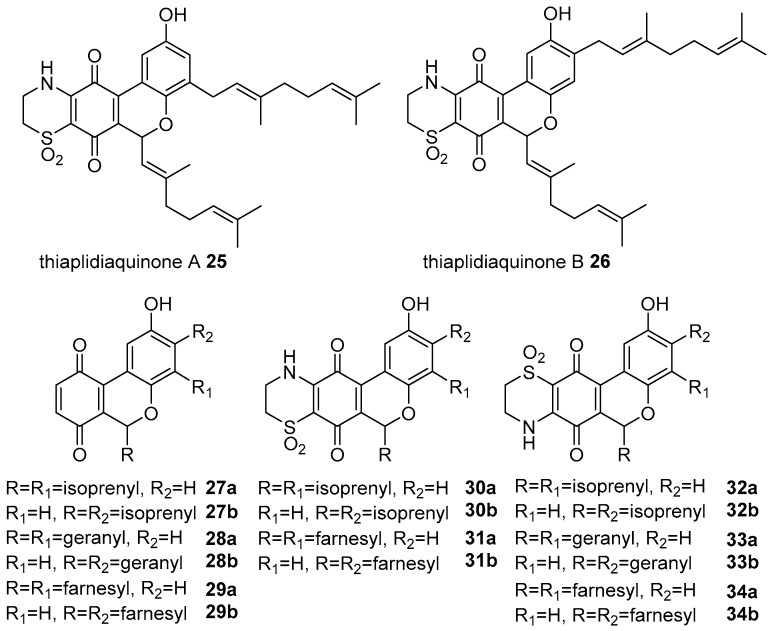
Structures of thiaplidiaquinones A, B and several synthetic analogues.

Other meroterpenoids containing a benzo[*c*]chromene-7,10-dione scaffold were thiaplidiaquinones A and B (**25** and **26**) (Figure 4), which bore an additional dihydrothiazine-fused ring. Since they were reported in 2005 by Fattorusso et al. [15], several studies dealing either with their synthesis or their biological evaluation had been published. They were isolated from the Mediterranean ascidian *Aplidium conicum*, collected at Sardinia (Italy) and almost simultaneously, interesting biomimetic syntheses of thiaplidiaquinones were published by Moody et al. [16] and Copp et al. [17]. Moody used a facile oxa-6π-electrocyclic ring closure reaction starting from simple phenolic precursors, followed by installation of the 1,4-thiazine-dioxide ring by reaction with hypotaurine, while Copp used a base-mediated dimerization of geranylbenzoquinone, followed also by addition of hypotaurine.

The thiaplidiaquinones A and B (**25** and **26**) were initially described as cytotoxic, inducing apoptosis by a mechanism that involved the production of intracellular reactive oxygen species (ROS) [15]. More recent studies showed that these natural compounds were weak inducers of ROS in Jurkat cells, while the synthetic precursors **28** and the dioxothiazine regioisomers **33** were more potent inducers [18] (Figure 4). The non-natural regioisomers were also more potent in the US-NCI tumor cells panel, with selectivity towards melanoma cell lines, and more active in antiplasmodial assays than the natural compounds. In the same study, it was found that the positioning of the geranyl side chain influences the mechanism of Jurkat cell death. Thus, analogues of thiaplidiaquinone A caused death dominantly by necrosis, while thiaplidiaquinone B analogues caused cell death via apoptosis [18]. The same authors synthesized a series of analogues (**27**–**34**) (Figure 4) modifying the length of the prenyl chains. In fact, they obtained isoprenyl and farnesyl series and compared their bioactivity with the geranyl series. They found that isoprenyl and farnesyl series were more potent than the geranyl series against *P. falciparum*; however, the geranyl series were better as inhibitors of the farnesyltransferase enzyme [19].

**Figure 5:**
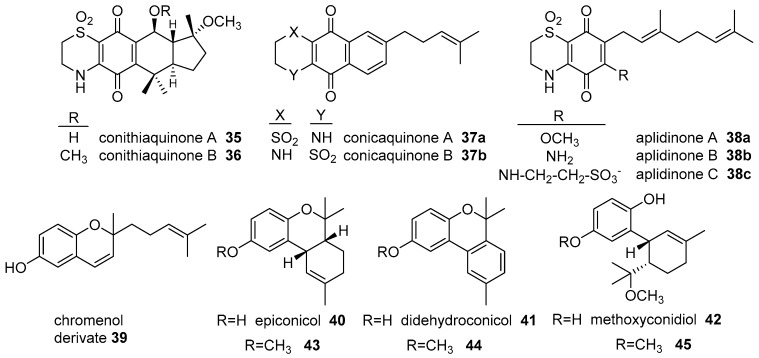
Structures of meroterpenoids **35**–**45.**

From the same ascidian *A. conicum* collected at Porto Cesareo, Italy, two thiazinoquinones were isolated, conithiaquinones A and B (**35** and **36**) (Figure 5). They have an uncommon linearly 6,6,5-ring skeleton with a dioxothiazine-fused ring, and showed an interesting cytotoxicity against MCF-7 human breast cancer cells. Other known meroterpenoids isolated from this species were conicaquinones A and B (**37a** and **37b**), and chromenols **39** and **40** [20] (Figure 5). To deepen into the mechanism of action of these secondary metabolites, four thiazinoquinones: thiaplidiaquinone B (**26**), conithiaquinones A and B (**35** and **36**), and conicaquinone A (**37a**) were subjected to voltammetric studies, with the finding that all of them were able to form semiquinone radical species which, depending on the protonation state, could be reduced or oxidized in the presence of redox-active components such as O_2_ and ROS, suggesting that their cytotoxic potency would be related to their ability for reduction and generation of radical species [21]. The same authors related not only this ability to form semiquinone species, but also a strong interaction with free Fe(III)-protoporphyrin IX with the high antiplasmodial activity showed by several alkyl analogues of aplidinones A–C (**38a**–**c**), which were other thiazinoquinones with a geranyl chain, previously isolated from *A. conicum* [2,22].

Epiconicol (**40**) was first isolated from the ascidian *Aplidium aff. densum*, together with didehydroconicol (**41**) and methoxyconidiol (**42**) (Figure 5), and they are examples in which the original geranyl chain was cyclized to a menthane [23]. These compounds and their monomethylated derivatives **43**–**45** (Figure 5) were easily synthesized from 1,4-HQ or 4-methoxyphenol and citral, and then they were evaluated as antibacterial against *Escherichia coli* and *Micrococcus luteus*, and as an antiproliferative against sea urchin eggs and a panel of cancerous and normal cells [24]. It was found that only **40** and **41** were weakly active in the antibacterial assays. In the antiproliferative assays, methoxyconidiol (**42**) showed the best results at the μM level, while the methylation of a phenolic group produced a total loss of activity in all of them [24].

**Figure 6:**
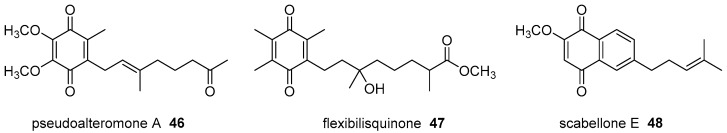
Structures of the quinone meroterpenoids **46**–**48.**

Pseudoalteromone A (**46**) (Figure 6), was a new ubiquinone analogue isolated from a marine bacterium *Pseudoalteromonas* sp., originally isolated from a cultured-type octocoral *Lobophytum crassum*, from the National Museum of Marine Biology and Aquarium in Taiwan. It had an uncommon nor-monoterpenoid moiety, probably formed by degradative oxidation of a methylidene group in the monoterpenoid chain. It exhibited significant cytotoxicity towards four tumoral cell lines (IC_50_ = 3.7–9.5 μg/mL), and also displayed a significant inhibitory effect on the elastase release by human neutrophils, an assay to predict anti-inflammatory activity [25].

Studies on the cultured soft corals *Sinularia flexibilis* and *Lobophytum hsiehi* from the same Museum in Taiwan, led to the isolation of flexibilisquinone (**47**) (Figure 6), another ubiquinone analogue with a methyl ester function on the monoterpenoid moiety [26]. It also displayed in vitro anti-inflammatory effect by inhibiting the accumulation of the pro-inflammatory iNOS and COX-2 proteins of the LPS-stimulated RAW264.7 macrophage cells [26].

Not only BQ meroterpenoids have been identified from marine organisms, but there are also diverse NQ derivatives. One simple example of this kind is scabellone E (**48**) (Figure 6), a NQ derivative that was first obtained during the biomimetic cyclization of 2-geranyl-6-methoxyhydroquinone, and which was identified later as a natural compound in the extract of *A. scabellum* [14].

**Figure 7:**
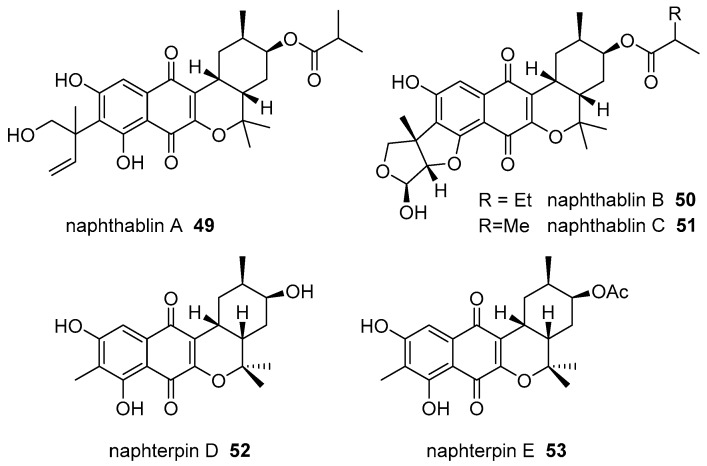
Structures of naphthablins and naphterpins **49**–**53**.

Other NQ meroterpenoids are naphthablins A–C (**49**–**51**) (Figure 7), which were identified from a marine strain of actinomycete, *Streptomyces* sp. CP26-58, isolated from the marine sediment collected at the San Francisco Bay, California. They exhibited weak HeLa cell growth inhibition [27]. From a very close marine strain *Streptomyces* sp. CNQ-509 isolated also from a marine sediment collected off La Jolla, California, two new NQ-based meroterpenoids were identified and named naphterpins D and E (**52**–**53**) (Figure 7). In these NQs, the monoterpenoid chain was modified by cyclization and hydroxylation. Compounds **52** and **53** exhibited free radical scavenging activity [28].

**Figure 8:**
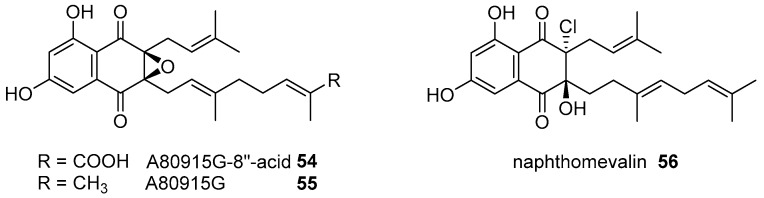
Structure of meroterpenoids **54**–**56**.

**Figure 9:**
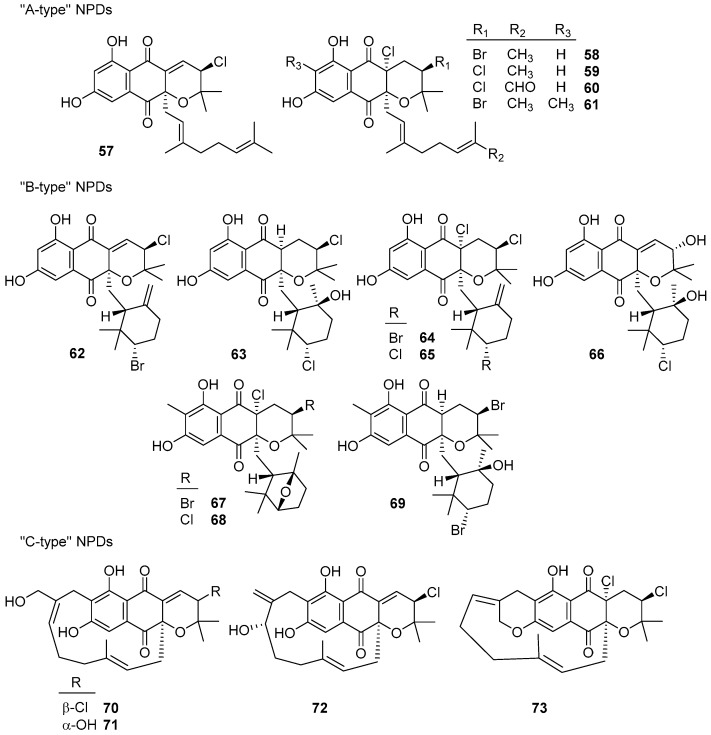
Structures of napyradiomycins (NPDs) **57**–**73** from marine *Streptomyces* sp.

A NQ derivative with geranyl and isoprenyl chains, A80915G-8′′-acid **54** (Figure 8), was identified from the *Streptomyces* sp. MS239, a marine-derived *Streptomyces* strain isolated from saline mud collected at Tokushima, Japan, that presented mevalonate pathway genes. This compound showed weak antibacterial activity against *Bacillus subtilis* (MIC 64 μg/mL) and it was inactive to *Staphylococcus aureus* and *Enterococcus faecium* [29]. In extracts from the same strain, other related known meroterpenoids such as A80915G (**55**), naphthomevalin (**56**) (Figure 8), and napyradiomycins A1 and B1 (**59** and **65**) (Figure 9) were also identified. These halogenated meroterpenoids were prenylated at C-2 and C-3 of the naphthalene core, which was an unusual substitution pattern from a biosynthetic point of view; only recently have Moore et al. reported two enzymes that are responsible for the prenylation and an α-hydroxyketone rearrangement that explained such a pattern [30,31]. The same authors performed a biomimetic total synthesis of naphthomevalin via a thermal α-hydroxyketone rearrangement, which supported the pathway [30].

Napyradiomycins (NPD) are a family of natural halogenated meroterpenoids that are produced by diverse marine-derived *Streptomyces* strains. These natural secondary metabolites bear an isoprenyl and a geranyl chain on the NQ core, forming additional cycles. Since the isolation of the first NPD in the 1980s from the actinomycete *Chainia rubra*, which was later reclassified as *Streptomyces ruber*, nearly 50 different NPDs have been described [32]. The nomenclature used for these compounds in the literature is very complex, and sometimes includes the strain number and molecular weights, and sometimes other numbers and letters, etc. that make it difficult to follow them properly. All of the NPDs have the isoprenyl unit cyclized on the NQ core, forming an additional benzochromene system. The monoterpenoid chain was a linear substituent in the “A-type” NPDs, a cyclic monoterpenoid substituent in “B-type” NPDs, and it constitutes a monoterpenoid bridge between both rings of the NQ core, forming a 14-membered ring in “C-type” NPDs (Figure 9).

From the marine-derived *Streptomyces* sp. SCSIO 10428, isolated from a sediment sample collected from Xieyang Island, China, two new A-type NPDs **57** and **58** were isolated, along with the known A-type NPDs **59** and **60**, two known B-type NPDs **64** and **65**, one known C-type NPD **72** (Figure 9), the known naphthomevalin **56** (Figure 8), and 3-chloro-6,8-dihydroxy-α-lapachone (**9**) (Figure 1), mentioned above [11]. NPDs **57**, **59** and 3-chloro-6,8-dihydroxy-α-lapachone were also isolated from a cultured marine-derived *Streptomyces* sp. CA-271078, isolated from an ascidian collected at Sao Tome, Sao Tome and Principe, along with a new B-type NPD **66 [11,32]** (Figure 9). Two B-type NPDs **62** and **63** and four C-type NPDs **70**–**73** (Figure 9) were identified from two marine-derived *Streptomyces* sp. CNQ-329 and CNH-070 isolated from a marine sediment sample collected at San Diego, California [33], while the strain CNQ-525 isolated from a similar marine sediment sample collected at La Jolla, California, yielded one brominated A-type NPD **61** and three B-type NPDs, **67**–**69** [34] (Figure 9).

In general, NPDs exhibit diverse bioactivities, particularly antibacterial. Most of the NPDs described above demonstrated potent bactericidal activity against contemporary methicillin-resistant *S. aureus* strains, particularly those of the A- and B-type, being those of the C-type are less potent compounds [11,32,33,35]. Some of them were tested for their antifungal activity, although none of them were active [32], and also for their cytotoxicity against several tumor cell lines, with the finding that they were cytotoxic at the μM level, being able to induce apoptosis in a dose-dependent manner [11,33,34].

## 4. Meroterpenoids with Open-Chain and Cyclic Sesquiterpenoids

Meroterpenoids that incorporate a sesquiterpenoid moiety are a wider group of TQs/THQs from marine origins. The sesquiterpenoid can be open-chain or can undergo intramolecular cyclization and/or rearrangements of the original farnesyl chain to form either monocyclic or polycyclic cores. Thus, they can have a wide variety of sesquiterpenoid skeletons.

### 4.1. Open-Chain Sesquiterpenoids

In this section, compounds with a non-cyclic farnesyl chain are considered together with the corresponding chromene derivatives resulting from the cyclization of the acyclic sesquiterpenoid on the aromatic fragment.

**Figure 10:**
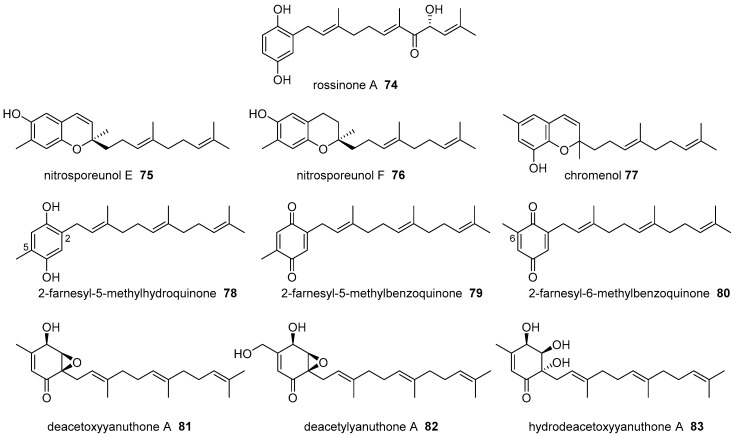
Structures of meroterpenoids with acyclic sesquiterpenoids **74**–**83**.

From an ascidian *Aplidium* sp. collected at Ross Sea, Antarctica, an oxygenated derivative of the farnesyl HQ, rossinone A (**74**) (Figure 10), was isolated. It showed in vitro anti-inflammatory activity acting as suppressor of superoxide production rather than as superoxide scavenger; it also exhibited modest antimicrobial, antifungal, and antiviral activities against *B. subtilis*, *Trichophyton mentagrophytes*, and HSV-1 respectively [8].

Two new chromenol derivatives, nitrosporeunols E and F (**75** and **76**), were isolated from the mutated marine-derived strain of Arctic *Streptomyces nitrosporeus* YBH10-5, along with the known 2-farnesyl-5-methylhydroquinone (**78**), 2-farnesyl-5-methylbenzoquinone (**79**), and deacetoxyyanuthone A (**81**) (Figure 10). They were tested as antihyperlipidemics, and it was found that only **79** was a potent lipid-lowering agent, which decreased lipid levels through upregulation of the PARPα pathway in HepG2 cells. This kind of activity is rarely reported for extracts of marine microorganisms [36]. Deacetoxyyanuthone A (**81**) was also found in the fungus *Gliomastix* sp. ZSDS-F7, isolated from the sponge *Phakellia fusca* from Yongxing Island of Xisha, along with deacetylyanuthone A (**82**) and hydrodeacetoxyyanuthone A (**83**) (Figure 10). They showed significant cytotoxicity against different cancer cell lines and, in addition, hydrodeacetoxyyanuthone A (**83**) exhibited antimycobacterial activity [37].

The regioisomer of **79**, 2-farnesyl-6-methylbenzoquinone (**80**), and the new chromenol **77** were isolated from the brown alga *Homoeostrichus formosana*, collected off the coast of San-Hsian-Tai, Taiwan. They had moderate cytotoxicity against three cancer cell lines, and good antibacterial activity against *Pseudomonas aeruginosa*; in addition, farnesylmethylbenzoquinone **80** was found to display potent in vitro anti-inflammatory activity by inhibiting superoxide anion formation [38].

**Figure 11:**
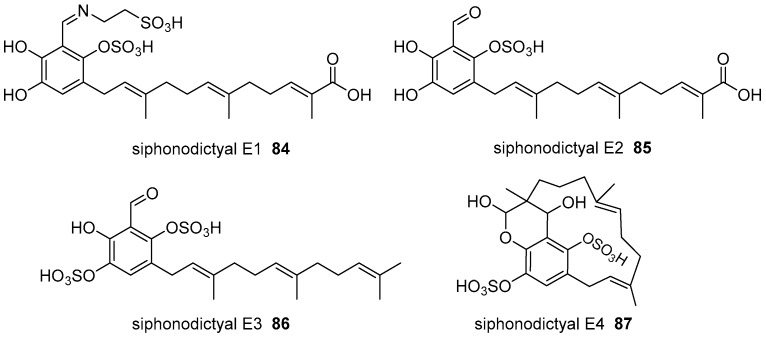
Structures of siphonodictyals E1–E4 (**84**–**87**) from *A. coralliphagum.*

New sulfated HQs, siphonodictyals E1–E4 (**84**–**87**) (Figure 11), were isolated from the Caribbean sponge *Aka coralliphagum* collected off the coast of San Salvador, Bahamas. Compounds **84** and **85** bore the sesquiterpene chain oxidized at one of the terminal methyl groups, and **86** had two sulfated ester groups. For siphonodictyal E4 (**87**), the structure shown in Figure 10 was proposed, although it could not be unambiguously assigned. In addition to a chromane, it contained a 15-membered macrocycle probably formed by an aldol addition from a precursor with an acyclic sesquiterpenoid that was partially oxidized at its end. All four derivatives were evaluated for their cytotoxicity and antibacterial activities, and it was found that only **86** showed mild activity against Gram-positive bacteria, and none of them were cytotoxic, neither did any have any effects on Gram-negative bacteria [39].

**Figure 12:**
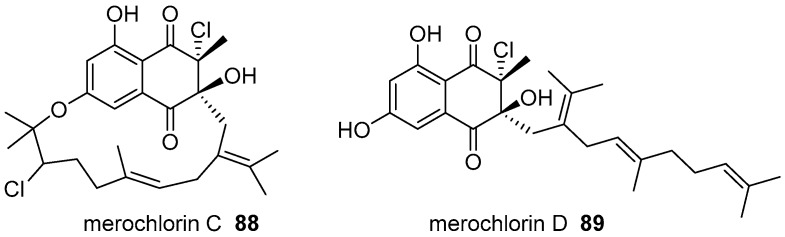
Structures of merochlorins C and D (**88** and **89**).

Only two examples of meroterpenoids with an open-chain sesquiterpenoid attached to a NQ core have been described in this period. They are merochlorins C and D (**88** and **89**) (Figure 12), produced by the marine bacterium *Streptomyces* sp. strain CNH-189, isolated from a marine sediment sample collected near Oceanside, California [40]. They had a sesquilavandulyl skeleton, an unusual rearranged C_15_-isoprene unit that is present in bacteria, whose biosynthesis was mediated by a vanadium-dependent chloroperoxidase Mcl24 that has been recently characterized [30].

There were several methylated toluquinols, cystoazorones [41] and cystodiones [42], isolated from brown algae of genus *Cystoseira* that had a 14-carbon chain. Initially, they seemed to be nor-sesquiterpenoids, however, it is more probably that they came from the degradation of a diterpenoid unit, as suggested by the authors and so, they are presented later in Section 5.

### 4.2. Monocyclic Sesquiterpenoids

**Figure 13:**
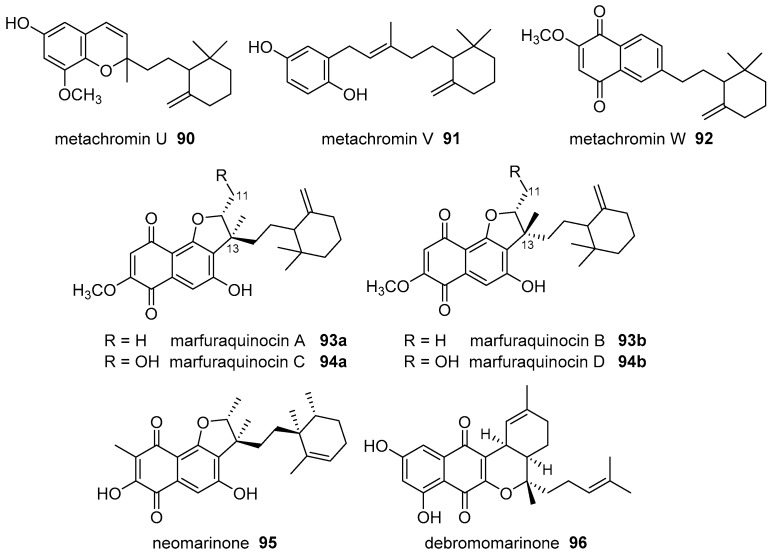
Structures of metachromins, marfuraquinocins, and marinones derivatives.

In the period covered by this review, few meroterpenoids with a monocyclic sesquiterpenoid unit were described. That was the case of metachromins U, V, and W (**90**–**92**) (Figure 13), isolated from the marine sponge *Thorecta reticulate* collected off Hunter Island, Tasmania. All three compounds had the same terminal cyclohexane moiety, but they differed at the other end of the sesquiterpenoid. While **90** was a chromene derivative, **91** was a HQ and **92** had an additional C–C bond between the aromatic part and the terpenoid forming a NQ moiety. Metachromins U-W were found to be cytotoxic against several cancer cell lines, with **91** being the most potent and **92** the less active [43]. Other previous metachromins were compiled in the chapter of de Rosa and Tommonaro [1].

From the marine-derived *Streptomyces niveus* SCSIO 3406 isolated from a South China Sea sediment, several sesquiterpenoid NQs were identified and named marfuraquinocins A–D (**93**–**94**) (Figure 13), which differ in the stereochemistry at C-13 and in presence or absence of a hydroxyl group at C-11. All of them showed significant cytotoxic and antibacterial activities [44]. Another cytotoxic and antibacterial furonaphthoquinone with a different rearranged monocyclic sesquiterpenoid side chain is neomarinone (**95**) (Figure 13); it was isolated previously from a fermentation broth of the marine-derived actinomycete CNH-099, but its stereochemistry remained unknown until its total synthesis in 2009 by Pérez-Sestelo et al. [45]. Another member of the marinone family with a different cyclization pattern on the sesquiterpenoid moiety is debromomarinone (**96**) (Figure 13), which while known since the 1990s, it has been recently isolated from the marine strain of actinobacterium *Streptomyces* sp. CNQ-509 [28].

**Figure 14:**
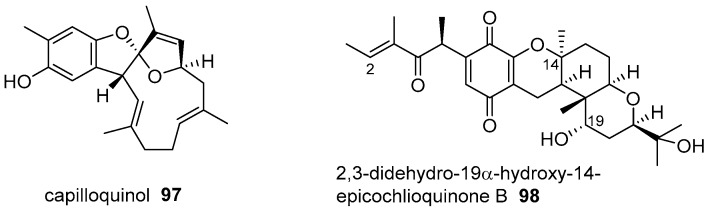
Structures of merosesquiterpenoids **97** and **98**.

Capilloquinol (**97**) (Figure 14) is a HQ with an unprecedented skeleton isolated from the soft coral *Sinularia capillosa* collected at Dongsha Atoll, Taiwan. It had a 12-membered ketalized macrocycle derived from the farnesyl chain. It was cytotoxic against the mouse leukemia P-388 but it did not show appreciable cytotoxicity against A-549 and HT-29 cells, nor was it active against human pathogenic cytomegalovirus [46].

A different cyclization pattern was found in 2,3-didehydro-19α-hydroxy-14-epicochlioquinone B (**98**) (Figure 14), in which a monocyclic terpenoid configured a to tetracyclic system that included two fused chromenoid systems. It was isolated from the fungal strain *Nigrospora* sp. MA75, obtained from the Chinese marine semi-mangrove plant *Pongamia pinnata*, and it showed antibacterial and cytotoxic activities [47].

### 4.3. Bicyclic Sesquiterpenoids

**Figure 15:**
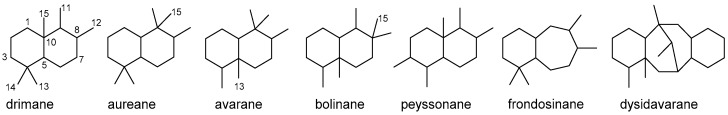
Bicyclic and polycyclic sesquiterpene skeletons in marine products.

This is the largest group of merosesquiterpenoids that are considered in this review. Most of them have a bicyclic terpenoid, derived from a drimane or a rearranged drimane skeleton. Marcos et al. [6] performed a good compilation of this type of compounds and proposed the name of aureane and avarane for those that biogenetically arise from the drimane skeleton by one and two methyl migrations. Later, de Rosa and Tommonaro [1] added the names bolinane, peyssonane, and frondosinane for compounds with different rearrangements of the sesquiterpenoid skeleton, and also Jiao et al. [48] proposed the name of dysidavarane for an unprecedented carbon skeleton, which includes all the terpene and quinone atoms of the whole meroterpenoid. These skeletons are shown in Figure 15.

Compounds considered in this part have been organized according to their sesquiterpenoid skeletons, and they are subdivided, whenever possible, in an order of increasing substitution and complexity of the aromatic moiety. We should bear in mind that sometimes more than one type was found in the same species; thus, the same article may have been referenced more than once. Those compounds appearing in previous reviews were not considered herein unless some new bioactivity or synthesis was described.

Thus, the compounds are organized in four subsections: drimane derivatives, aureane derivatives, avarane derivatives, and other cyclic merosesquiterpenoids. Mainly, the polyketide fractions are BQ/HQs, but sometimes they can be partially reduced or even etherified, and/or form additional bonds between both fragments. In this sense, those compounds having relative 1,2- or 1,4-oxigenated functions at this polyketide core are considered here.

#### 4.3.1. Drimane Skeleton

The meroterpenoids described here were formed by a drimane skeleton attached to a BQ/HQ system, and they were isolated from sponges, algae, and marine-derived fungi.

**Figure 16:**
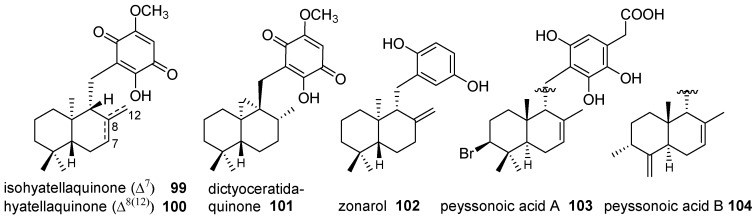
Structures of drimane sesqui-terpenyl-quinones/hydroquinones (TQs/THQs) **99**–**104**.

Isohyatellaquinone (**99**) (Figure 16) is a sesquiterpenoid quinone isolated from the sponge *Dactylospongia elegans* collected at the Inner Gneering Shoals near Mooloolaba, Australia [49]. It was isolated along with the known drimane derivatives hyatellaquinone (**100**) and dictyoceratidaquinone (**101**) (Figure 16). The relative stereochemistry of the latter was assigned in that work. Other sesquiterpenoid quinones, isolated from the same specimen, were mamanuthaquinone and ilimaquinone, which had a different sesquiterpene skeleton [49].

Zonarol (**102**) (Figure 16) is a well-known sesquiterpene HQ that was isolated from the brown alga *Dictyopteris undulata*, and it was described originally as fungitoxic [50], later as anti-inflammatory [51] and, more recently, as a protector against dextran sodium sulfate-induced colon injury in mice (a model of the inflammatory bowel disease ulcerative colitis) [52]. In addition, it was found that zonarol activated the Nrf2/ARE pathway and protected neuronal cells against oxidative stress, this being the first time that such activity was reported for any compound from brown algae [53].

Lane et al. evaluated the antimicrobial defences for 69 collections of Fijian red macroalgae against three microbial pathogens of marine macrophytes, and found that some members of the red macroalga genus *Peyssonnelia* showed strong antimicrobial activity [54]. Bioassay-guided chromatography led to the isolation of peyssonoic acid A (**103**) and peyssonoic acid B (**104**) (Figure 16). They are HQ meroterpenoids attached to acetic acid; peyssonoic acid A (**103**) had a brominated drimane decaline, whereas peyssonoic acid B (**104**) had a rearranged peyssonane terpenoid. Both inhibited the growth of two marine alga pathogens, the bacteria *Pseudoalteromonas bacteriolytica*, and the fungi *Lindra thalassiae*, and showed modest antineoplastic activity against ovarian cancer cells [54].

There are several meroterpenoids with a cyclohexenone/cyclohexenedione core attached to the sesquiterpenoid moiety, and although they are not proper quinones, they are structurally related, having the same polyketide nature, and as they have oxygen functions in 1,2-/1,4-relative positions, they have been considered as partially reduced or modified quinones, and so they are included in this revision.

**Figure 17:**
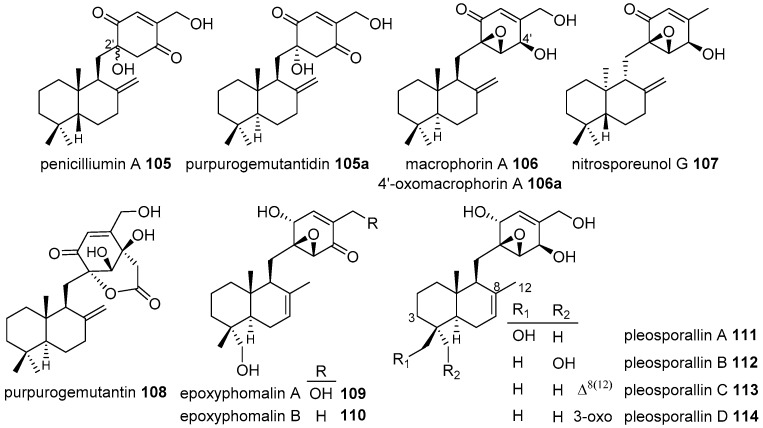
Structures of merosesquiterpenoids **105**–**114** from several fungi and bacteria.

At the beginning of 2012, Lin et al. published the isolation of a new sesquiterpene quinone, named penicilliumin A (**105**) (Figure 17), from a new fungal strain *Penicillium* sp. F00120, isolated from a deep-sea sediment sample collected in the northern South China Sea [55]. This compound inhibited in vitro proliferation of mouse melanoma B16, human melanoma A375 and HeLa cells moderately. The authors assigned the relative stereochemistry of the drimenyl core, but they did not determine the absolute configuration at the position C-2′ [55]. A few months later, Fang et al. isolated a drimenyl cyclohexenone derivative that had the same ^1^H and ^13^C data, but that differed in physical appearance and optical rotation though with the same sign [56]; this came from the bioactive mutant BD-1-6 of a marine-derived *Penicillium purpurogenum* G-59. These facts, together with the predicted and calculated CD spectra, served to assign the structure **105a** and claimed it as a new compound with the name purpurogemutantidin. From the same mutant strain, another new metabolite, purpurogemutantin (**108**), and the known macrophorin A (**106**) were also isolated and characterized (Figure 17). None of them were produced by the parent strain, but all of them showed significant antiproliferative effect against several human tumor cell lines [56]. Purpurogemutantin (**108**), macrophorin A (**106**), and 4′-oxomacrophorin A (**106a**) were also found in the fungus *Gliomastix* sp. ZSDS1-F7, isolated from the sponge *Phakellia fusca* from Yongxing Island of Xisha, along with several yanuthone A analogues that have been mentioned above (Figure 10). According the authors, this was the first time that meroterpenoids with both open-chain, and bicyclic drimane skeletons were reported from the same fungal strain [37]. An example of both types of meroterpenoids in bacteria is the mutated marine-derived strain of Arctic *Streptomyces nitrosporeus* YBH10-5, from which nitrosporeunol G (**107**), a meroterpenoid related to macrophorin A, was described as a new compound, along with several farnesylquinones [36].

From the marine-derived fungus *Paraconiothyrium* sp. (former *Phoma* sp.), obtained from the marine sponge *Ectyplasia perox* collected at Caribbean Sea, Dominica, two cyclohexanone derivatives epoxyphomalins A (**109**) and B (**110**) (Figure 17) were isolated, and they showed highly potent cytotoxicity on 12 out of 36 human tumor cell lines. They exerted their effect through inhibition of the 20S proteasome, enzymes responsible for the degradation of endogenous proteins [57,58].

Pleosporallins A–D (**111**–**114**) (Figure 17) are meroterpenoids isolated from *Pleosporales* sp. a fungus obtained from the marine alga *Enteromorpha clathrate*, collected at Monkey Island in the South China Sea. Pleosporallin D (**114**) exhibited moderate antibacterial activity and pleosporallins A-C (**111**–**113**), showed anti-inflammatory activity with no detectable cytotoxic effects [59].

**Figure 18:**
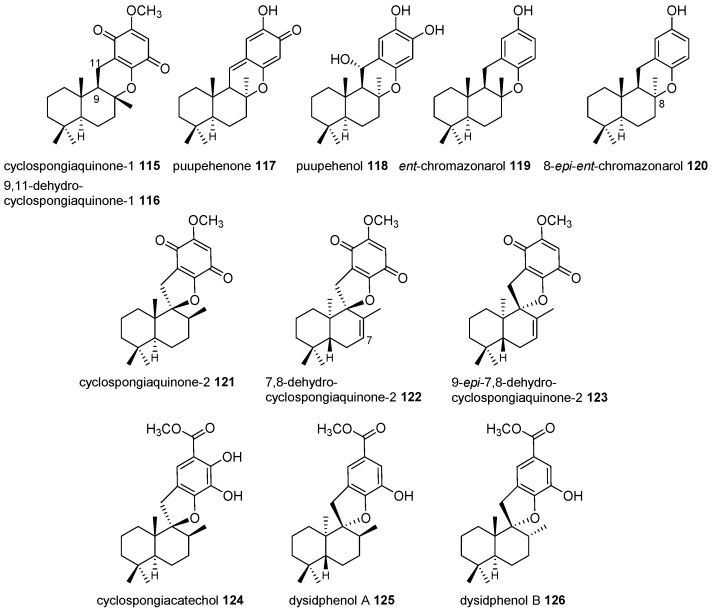
Structures of the merosesquiterpenoids **115**–**126** with oxygen bridge.

In this section, several sesquiterpenequinones/HQs with an additional oxygen bridge between the decaline and the polyketide ring are considered. This type of additional bond results in the formation of new fused-pyran or furan rings, which were the objective of many synthetic studies toward the total synthesis of these interesting bioactive metabolites. Many of them have been known for a long time, but some new synthetic procedures are being reported. This is the case for (+)-cyclospongiaquinone-1 (**115**) and (−)-dehydrocyclospongiaquinone-1 (**116**), which were synthesized from (+)-sclareolide by Takeda et al. [60], and several works on puupehenone (**117**), related compounds [61,62], or a nature-inspired synthesis of (+)-*ent*-chromazonarol (**119**) [63] (Figure 18).

Puupehenol (**118**) (Figure 18) was described as a new natural compound from a deep-water Hawaiian sponge *Dactylospongia* sp. along with the known puupehenone (**117**), suggesting that the latter may always be an artefact of the isolation procedures. Both compounds were very effective antioxidants and antimicrobials towards Gram-positive bacteria [64].

Two new spirane compounds 7,8-dehydrocyclospongiaquinone-2 (**122**) and 9-*epi*-7,8-dehydrocyclospongiaquinone-2 (**123**) (Figure 18) were isolated, along with other sesquiterpenoid quinones mentioned above, from the sponge *Dactylospongia elegans* collected at the Inner Gneering Shoals near Mooloolaba, Australia [49]. From the same species *D. elegans*, but collected off the coast of Palau and obtained from the US NCI’s Open Repository Program, several meroterpenoid quinones with diverse sesquiterpene skeletons were also isolated. Among those with a drimenyl moiety were 8-*epi*-*ent*-chromazonarol (**120**), cyclospongiaquinone-1 (**115**), cyclospongiaquinone-2 (**121**), and the new HQ cyclospongiacatechol (**124**) [65] (Figure 18).

Dysidphenols A and B (**125** and **126**) (Figure 18), are also spiranic meroterpenoids probably derived from protocatechuic acid. They were isolated from a purple-colored encrusting *Dysidea* sp. off the coral-reef near Yongxing Island in the South China Sea, and showed weak antibacterial activity against *E. coli*, *B. subtilis* and *S. aureus* [66].

#### 4.3.2. Aureane Skeleton

The meroterpenoids described here are formed by an aureane skeleton attached to a *p*-BQ or to a HQ, sometimes forming an additional pyran ring between the two fragments. The aureane skeleton is less frequent than its analogues, and it could be considered a biogenetically intermediate between drimane and avarane skeletons through a 1,2-methyl shift.

**Figure 19:**
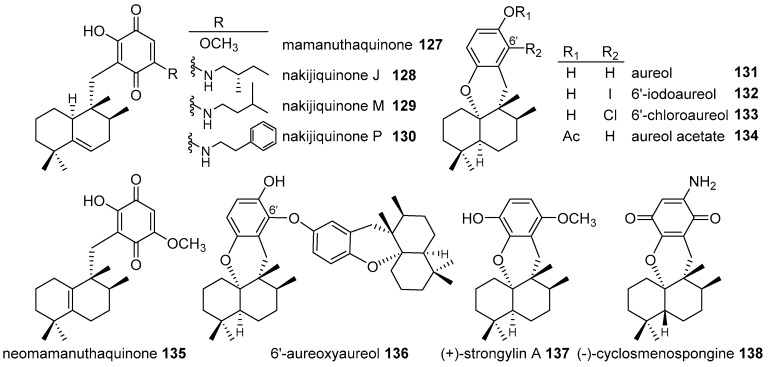
Structures of meroterpenoids with an aureane skeleton **127**–**138**.

A sponge of the family Spongiidae, SS-1047, collected off Gesashi, Okinawa (Japan) produced many aureane and avarane-derived sesquiterpenoidquinones with amine residues on the quinone ring. Among those having an aureanyl group, were nakijiquinones J, M and P (**128**–**130**) (Figure 19). Nakijiquinone P (**130**) exhibited a certain inhibitory activity against EGFR (epidermal growth factor receptor) tyrosine kinase (76% inhibition at 1 mM) [67].

The first iodo-sesquiterpenyl-HQ reported as a natural compound, 6′-iodoaureol (**132**), was isolated from the sponge *Smenospongia* sp. collected at PP Island, Thailand, along with the new sesqui-THQ dimer 6′-aureoxyaureol (**136**) [68]. In the same study, other aureanyl-HQs identified with an ether linkage between both fragments were aureol (**131**), 6′-chloroaureol (**133**) and aureol acetate (**134**) (Figure 19). The drimane derivative *ent*-chromazonarol (**119**) (Figure 18) was also identified.

*D. elegans* was collected at the Inner Gneering Shoals near Mooloolaba, Australia, and as mentioned above, it was also a source of meroterpenoids with an aureane skeleton, such as mamanuthaquinone (**127**) (Figure 19) [49]. From a different specimen, *Dactylospongia* sp., collected at the same place, and closely related to *D. elegans*, but likely to be new to science, neomamanuthaquinone (**135**) (Figure 19) was isolated as a new natural product, along with *ent*-isohyetallaquinone (*ent*-**99**) (Figure 16). Neomamanuthaquinone (**135**) was moderately active against breast cancer (BC), and it was inactive against small cell lung cancer NCI-H187 cell lines.

In this type of natural product, it is common to determine the molecular constitution and the relative stereochemistry, but the absolute configuration sometimes remains unassigned until the total synthesis is performed, either starting from an enantiomerically pure natural compound, or using stereocontrolled reactions. This is the case for (+)-strongylin A (**137**) [69], (+)-aureol (**131**) [70], and (−)-cyclosmenospongine (**138**) [71], which have been recently reported (Figure 19).

#### 4.3.3. Avarane Skeleton

Meroterpenoids with an avarane skeleton (also named 4,9-friedodrimane), are a larger group of these marine natural products that have been isolated mainly from sponges. They were found in both enantiomeric series, or epimerized at any position with a *trans*- or *cis*-fused decaline moiety. It is frequent that the same generic name with correlative letters is given for many of them, probably following the isolation order from the extracts, but independently of their structural features. Even, compounds with different sesquiterpene skeleton received the same generic name, making it difficult to follow and identify them. In addition, it was found that high variability in the amount of secondary metabolites in the same sponges, depending on the collecting zone, and even on the specific parts of their tissues (central part, peripheral zone, etc.), and only a few studies about the influence of those factors have been published [72,73].

**Figure 20:**
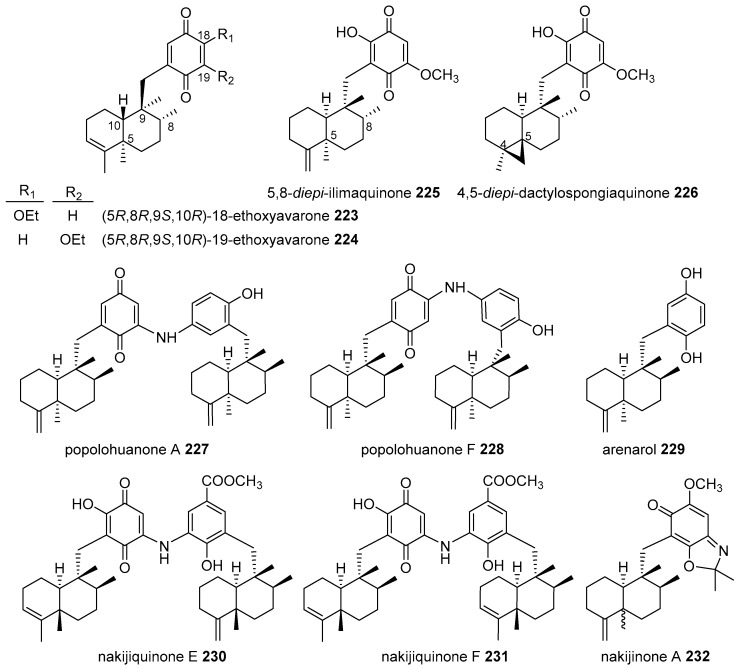
Structures of avarane-meroterpenoids not included in the above tables.

**Figure 21:**
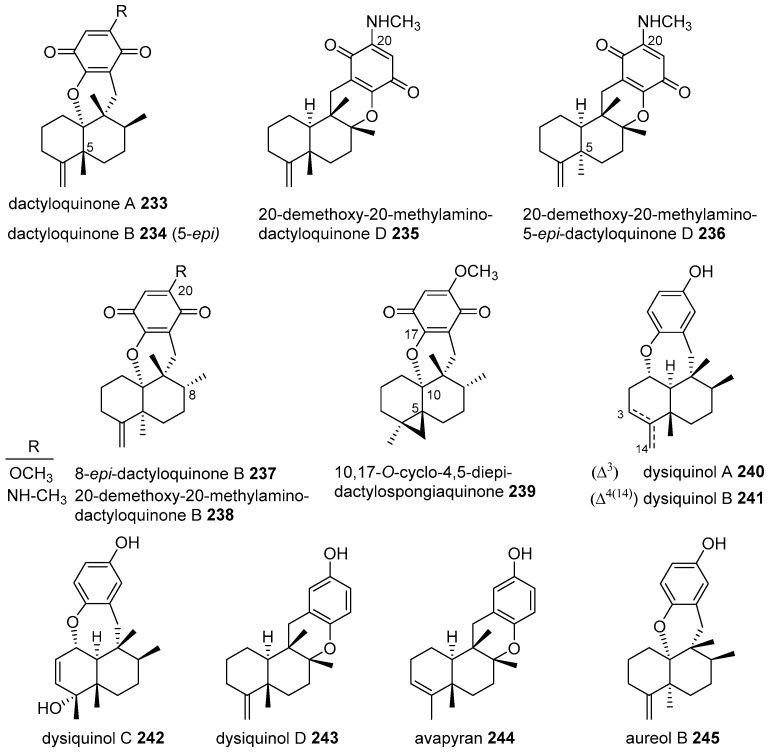
Structures of the avarane meroterpenoids with an epoxy bridge **233**–**245**.

Because more than one type or isomers were isolated from the same species, we presented the structures of all of them together at the beginning of this subsection, according to the structural similarities instead the chronological description, although afterwards, the discussion is based mainly on their source. Thus, the compounds were numbered correlatively in the tables by their structural similarities, but not by their appearance in the text. The compounds considered are shown in Table 1, Table 2, Table 3, Table 4, Table 5, Table 6 and Table 7 and Figure 20, and are classified as quinones and HQs, grouped according the *cis*- or *trans*-fusion in the decaline core and then, differentiating those with an endocyclic bond at C-3 from those with the exocyclic bond at C-4(14). There are also several compounds with oxygen bridges between both parts of the meroterpenoid forming additional pyran or oxepane rings, which are shown in Figure 21.

Most of the compounds included in this group were isolated from marine sponges and the first sesquiterpenequinone/HQ; Marcos et al. [6] gave the name “avarane” to the sesquiterpene skeleton for this reason. Avarone (**139**) (Table 1)/avarol (**196**) (Table 5) were isolated from the marine sponge *Dysidea avara* [74]. Since then, many other avarane TQs/THQs have been isolated from this and other marine sponge genus and species.

In fact, genus *Dysidea* is an abundant source of chemically diverse and biologically active meroterpenoids. In addition to avarol (**196**) (Table 5), which was isolated from specimens of *D. avara* collected at Xisha Islands in the South China Sea, (+)-18-ethoxyavarone (**223**) and (+)-19-ethoxyavarone (**224**) (Figure 20) were isolated, along with (−)-18-ethoxyneoavarone (**158**) and (−)-19-ethoxyneoavarone (**159**) (Table 2). Both pairs of compounds had a *trans*-decaline, but they belonged to the enantiomeric series; **223** and **224** are the only TQs, in this group, with their configurations shown in Figure 20. In the same study, the four new THQs dysiquinols A-D (**240**–**243**) (Figure 21) were also characterized. They had an oxygen bridge between the decaline and the aromatic core, forming a doubly fused pyran or oxepane ring [75]. They were tested against NCI-H929 melanoma cell line, and for their NF-κB inhibitory activity. All of them were cytotoxic, but only dysiquinol D (**243**) showed NF-κB inhibitory activity [75].

Amino-substituents on the quinone core were frequent in this meroterpenoid group. Thus, 13 new sesquiterpene aminoquinones, along with six known ones, were isolated from *Dysidea fragilis*, collected along the coast of Yongxing Island in the South China Sea. The new compounds called dysidaminones A-M (**141**–**147**, **160**–**165**) (Table 1 and Table 2) were evaluated for their cytotoxicity, and for NF-κB inhibitory activity. Preliminary structure-activity relationship analysis showed that compounds with the Δ^4(14)^ double bond in the decaline were much more potent on both activities than the others [76].

From *Dysidea villosa*, collected also at Yongxing Island in the South China Sea, several HQs with unprecedented pyridone substituents were isolated. They were called dysivillosins A-D (**200**–**203**) (Table 5), and they were evaluated for their anti-allergic activity. Interestingly, all four compounds could down-regulate the production of LTB_4_ and IL-4 in mast cells, with dysivillosin A (**200**) being the most potent agent [77].

The purple-coloured encrusting *Dysidea* sp. collected off the coral-reef near Yongxing Island in the South China Sea, mentioned above for being a source of drimane meroterpenoids, was also a source of avarane aminoquinone derivatives with a Δ^4(14)^ double bond in a *trans*-decaline: smenospongine (**176**), smenospongorine (**178**), smenospongiarine (**179**), smenospongidine (**180**), and the new derivative smenospongimine (**177**) (Table 3). All of them showed potent antibacterial activities [66].

From a *Dysidea* sp. collected at Scott Reef, Northwest Australia, several arenarone (**166**) (Table 2) derivatives were isolated along with the known compounds arenarol (**229**) and popolohuanone A (**227**), having *cis*-decalines (Figure 20). The new derivatives were 18-aminoarenarone (**167**), 19-aminoarenarone (**168**), 18-methylaminoarenarone (**169**), 19-methylaminoarenarone (**170**) (Table 2), and the dimeric popolohuanone F (**228**) (Figure 20). They were neither antibacterial nor cytotoxic, although popolohuanones A and F exhibited moderate potencies in scavenging the 2,2-diphenyl-1-picrylhydrazyl (DPPH) free radical [78].

From another *Dysidea* sp. collected at Chuuk Island in the Federal States of Micronesia, melemeleones C and D (**171** and **172**) (Table 2), containing a *cis*-decaline core and a taurine fragment, and aureol B (**245**) (Figure 21), a cyclic derivative of arenarol (**229**) (Figure 20), were isolated and evaluated as cytotoxic, antimicrobial, and inhibitory for Na^+^/K^+^-ATPase [79].

Three new avarol derivatives were isolated from a *Dysidea* sp. collected at Iriomote Island, Okinawa. They were named 17-*O*-acetylavarol (**198**), 17-*O*-acetylneoavarol (**199**) (Table 5) and avapyran (**244**) (Figure 21). In this study avarol (**196**), neoavarol (**197**) (Table 5), and 3′-aminoavarone (**140**) (Table 1) were also isolated. By acylation and methylation reactions, several semisynthetic derivatives of avarol and neoavarol were obtained and evaluated as inhibitors of protein tyrosine phosphatase 1B (PTP1B), an enzyme related to glucose metabolism. In general, the avarol-type meroterpenoids appeared to be better inhibitors than the neoavarol ones; however, 17-*O*-acetylneoavarol (**199**) was the most potent PTP1B inhibitor in this study [80].

A new sesquiterpenoid HQ, 19-hydroxypolyfibrospongol B (**208**) was isolated from the marine sponge *Dysidea arenaria* collected off Hainan Island in South China Sea, along with the known compounds polyfibrospongol B (**206**) (Table 6), ilimaquinone (**174**), and smenospongine (**176**) (Table 3). All were evaluated for their in vitro anti-HIV activity on HIV-RT, with the finding that polyfibrospongols were inactive, whereas the quinones displayed moderate inhibitory activity [81].

Kobayahsi et al. performed several chemical composition studies on collections of marine sponges of the family *Spongiidae* collected off the Unten Port, Kerama Islands, and Gesahi, Okinawa, from which many sesquiterpenoid quinones containing amino substituents were isolated and given the generic name of nakijiquinones. The first compounds of this family, nakijiquinones A–D were described before [82,83], and nakijiquinones E–S were isolated during the period covered by this review. Nakijiquinones E and F (**230** and **231**) (Figure 20) are pseudodimeric meroterpenoids from sponge SS-1037 that did not show cytotoxicity against murine leukemias P-388 and L1210, and KB human epidermoid carcinoma [84]. Nakijiquinones G–I (**153**–**155**) (Table 1) from collections SS-1074 and SS-1047, had residues of histamine, agmatine, and methylsulfinylpropanamine respectively, being modestly cytotoxic against the same cell lines. They exhibited inhibitory activity on HER2 kinase, and antimicrobial and antifungal activities [85]. From collections SS-1047, SS-265 and SS1208, nakijiquinones K, L, N, O, Q, and R, (**149**, **181**, **150**–**152** and **156**) (Table 1 and Table 3), were isolated, having 2-methylbutylamine, isopentylamine, isobutylamine, phenetylamine, and taurine residues attached to the quinone ring. Nakijiquinones J, M, and P have an aureane skeleton and nakijiquinones N, O, and R showed inhibitory activity against HER2 tyrosine kinase [67]. From collection SS-1202, nakijiquinone S (**148**) (Table 1), with a *n*-butoxy substituent, and the *o*-hydroquinone derivative nakijinol C (**204**) (Table 6) were isolated, displaying antimicrobial activities against several bacteria and fungi [86].

Almost simultaneously, the same generic names were used by Daletos et al. for different meroterpenoids isolated from the sponge *Dactylospongia metachromia*, collected at Ambon, Indonesia [87]. The sesquiterpene quinones isolated from *D. metachromia* had a *cis*-decaline, so that the authors used the 5-*epi*-nomenclature, even though the corresponding *trans* isomers were not known by the authors at that time. This has led to the fact that nakijiquinone S (**148**) (Table 1) and 5-*epi*-nakijiquinone S (**190**) (Table 4) do not differ only in the configuration at C-5, as it should be, but also in the aminosubstituent on the quinone ring. Even greater are the differences between nakijinol C (**204**) (Table 6) and 5-*epi*-nakijinol C (**221**) (Table 7), the latter having an oxazole ring fused to the quinone moiety. Other new sesquiterpenoid quinones isolated from *D. metachromia* were 5-*epi*-nakijiquinone Q (**189**), 5-*epi*-nakijiquinone T (**192**), 5-*epi*-nakijiquinone U (**191**), 5-*epi*-nakijiquinone N (**188**) (Table 4), and the oxazolobenzoxazole 5-*epi*-nakijinol D (**222**) and the known 18-hydroxy-5-*epi*-hyrtiophenol (**219**) (Table 7). All of the quinones showed pronounced cytotoxicity, while the benzoxazoles showed a significantly lower cytotoxicity; they were also tested on a panel of 16 protein kinases involved in the regulation of tumor growth and metastasis, with similar results [87].

The benzoxazole nakijinol B (**214**) (Table 7) was described earlier from *Dactylospongia elegans*, collected at the northeast of Truant Island, Australia, together with the new meroterpenoids smenospongine B (**183**) and smenospongine C (**184**), and the already-known ilimaquinone (**174**) (Table 3) and dactyloquinone B (**234**) (Figure 21) [88]. They were found to have moderate to good cytotoxic activities on a panel of human tumor cell lines [88]. From the same species *D. elegans*, though collected off the coast of Palau, as mentioned before, several meroterpenoid quinones with diverse sesquiterpene skeletons were also isolated. In addition to ilimaquinone (**174**), among those with an avaranyl moiety were: 5,8-*diepi*-ilimaquinone (**225**), 4,5-*diepi*-dactylospongiaquinone (**226**) (Figure 20), 8-*epi*-dactyloquinone B (**237**), and 10,17-*O*-cyclo-4,5-*diepi*-dactylospongiaquinone (**239**) (Figure 21) [65]. Compounds **237** and **239** displayed a quite a unique *trans* configuration for the methyl groups at C-8 and C-9. All of these compounds from *D. elegans* were cytotoxic, and in addition, ilimaquinone (**174**), 5,8-*diepi*-ilimaquinone (**225**), and 4,5-*diepi*-dactylospongiaquinone (**226**) activated hypoxia-inducible factor-1 (HIF-1), and increased the expression of HIF-1 target vascular endothelial growth factor (VEGF) gene in T47D cells, representing potential drug leads for the prevention and/or treatment of ischemic disorders [65]. The opposite effect of hypoxia-selective growth inhibitory activity against DU145 cells was described for HQ dictyoceratin A (smenospondiol, **210**) (Table 6), either for the natural product isolated from *D. elegans* [89], and for the two (+) and (−)-synthetic enantiomers [90].

From a marine sponge *Spongia* sp., collected off Son Cha, Vietnam, many sesquiterpene aminoquinones/HQs were isolated by Morita et al. [91,92,93]. The aminoquinones were called langcoquinones A-F (**177**, **185**, **175**, **186**, **187**, **182**) (Table 3), and the HQs were called langconols A–C (**211**–**213**) (Table 6). They were assessed for antibacterial activities, being inactive against Gram-negative bacteria but being selectively active on Gram-positive bacteria. Some of them also showed significant cytotoxicity against several human cancer cell lines. Other sesquiterpene quinones already known and isolated from this Vietnamese *Spongia* sp. were dictyoceratin A (**210**), polyfibrospongol A (**205**), polyfibrospongol B (**206**), 19-hydroxypolyfibrospongol B (**208**) (Table 6), ilimaquinone (**174**), smenospongine (**176**), smenospongidine (**180**), smenospongorine (**178**), and nakijiquinone L (**181**) (Table 3).

It is necessary to mention that, as has happened before, the same compound was isolated almost simultaneously from different sources and received different names. Thus, the first compound of the above family, langcoquinone A (**177**) (Table 3) [91], bearing a methylamino group, has the same structure given for smenospongimine, isolated from *Dysidea* sp. [66].

New avarane-HQs, named smenohaimiens C–E (**217**, **207**, **209**) (Table 7 and Table 6), were isolated from the sponge *Smenospongia cerebriformis*, along with polyfibrospongol A (**205**), polyfibrospongol B (**206**), 19-hydroxypolyfibrospongol B (**208**) (Table 6), and ilimaquinone (**174**) (Table 3) [94]. They were moderate inhibitors of nitric oxide production in lipopolysaccharide-stimulated BV2 microglia cells, obtaining the best results with ilimaquinone.

From the sponge *Spongia pertusa*, collected off the coast of Yongxing Island, China, several sesquiterpene quinones were described by the first time, either with *cis*- and *trans*-decalines, and even from the enantiomeric series such as *N*-methyl-*ent*-smenospongine (*ent*-**177**) (Table 3) and *N*-methyl-5-*epi*-smenospongine (**195**), along with the known 5-*epi*-smenospongine (**194**) (Table 4), smenospongine (**176**), and smenospongiadine (**180**) (Table 3). From the same sponge, new analogues with an additional pyran ring were also identified as 20-demethoxy-20-methylaminodactyloquinone D (**235**), 20-demethoxy-20-methylamino-5-*epi*-dactyloquinone D (**236**), and 20-demethoxy-20-methylaminodactyloquinone B (**238**) (Figure 21) [95].

It has been reported that ethylsmenoquinone (**173**) and ilimaquinone (**174**) (Table 3) exhibited antiproliferative activity on multiple myeloma cells by down-regulating the expression of the oncogenic protein β-catenin [96]. In other to obtain new derivatives with such property, new avarane sesquiterpenequinones were generated by biotransformations of the meroterpenoids typically isolated from *Smenospongia* sponges, using the strong oxidative potential of the sponge *Verongula rigida*. Thus, *Smenospongia aurea* and *S. cerebriformis* were homogenized and incubated with *V. rigida* generating several new meroterpenoids such as (+)-5-*epi*-20-*O*-ethylsmenoquinone (**193**) (Table 4) and the benzoxazoles (−)-nakijinol E (**215**), (+)-5-*epi*-nakijinol E (**216**) (Table 7), and a mixture of nakijinone A and 5-*epi*-nakijinone A (**232**) (Figure 20). As the accumulation of intracellular β-catenin is also important in the development and progression of colorectal cancer, they were evaluated against two β-catenin response transcription (CRT)-positive colon cancer cell lines, and it was found that they were able to suppress CRT by promoting the degradation of β-catenin, with (+)-5-*epi*-20-*O*-ethylsmenoquinone (**193**) (Table 4) being the most potent compound [97].

Nakijinols B (**214**) (Figure 21) and E (**215**) (Table 7) were also identified from a *Hyrtios* sp. sponge collected off Yongxing Island in the South China Sea, from which two new benzoxazole analogues named nakijinol F (**217**) and nakijinol G (**218**) (Table 7) were isolated, and it was found that nakijinol G (**218**) showed PTP1B inhibitory activity, but no cytotoxicity against four human cancer cell lines [98]. Again, the structure described for nakijinol F was identical to that of smenohaimien C isolated from the sponge *S. cerebriformis* [94]. Nakijinols A (**220**), B (**214**), and E–G (**215**, **217**, and **218**) (Table 7) were efficiently synthesized recently by Takeda et al., starting from smenospongine (**176**) (Table 3), with confirmation of their absolute configurations [99].

Several derivatives of ilimaquinone were also obtained through biotransformation in the presence of the mold fungus *Mucor circinelloides*, which induced a stereospecific β-epoxidation of the exocyclic Δ^4(14)^ double bond by the most hindered face, compared with chemical epoxidation that led to the α-isomer as the major one. Hydroxylation of the decaline at C-3 was also detected, as well as an unexpected substitution on the quinone ring by ethanolamine. None of these compounds showed antiproliferative effect against three human cancer cell lines [100].

Total synthesis of (+)-avarone (**139**) (Table 1), (+)-avarol (**196**) (Table 5), (−)-neoavarone (**157**) (Table 2), (−)-neoavarol (**197**) (Table 5), and (+)-aureol B (**245**) (Figure 21) was efficiently performed by Sakurai et al. from (+)-5-methyl-Wieland-Miescher ketone. In vitro cytotoxicity assays on human lymphoma cells U937 showed that quinone derivatives were more potent than the corresponding HQs, and also that the position of the olefinic double bond has a large effect on activity, being better those quinones with an exocyclic double bond in the decaline [101].

#### 4.3.4. Other Bicyclic Sesquiterpenoids

**Figure 22:**
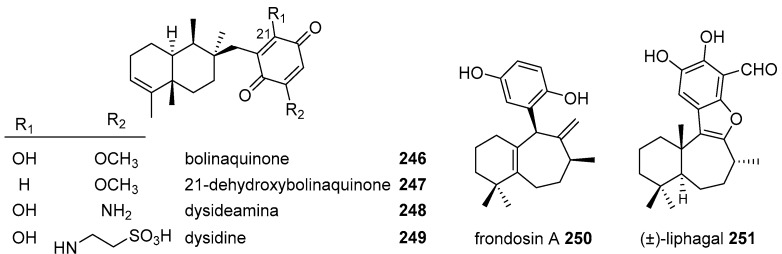
Structure of meroterpenoids with bolinane- and frondosinane-related skeletons.

Another sesquiterpenoid skeleton resulting from a different rearrangement of the initial drimane skeleton is bolinane. The representative TQ of this group is bolinaquinone (**246**) (Figure 22), isolated first in 1998 from a Philippine sponge *Dysidea* sp., and showing relevant anti-inflammatory properties by the inhibition of phospholipase (PL) A2 [102]. It significantly reduced the production of mediators in the inflammatory processes, and it modulated oxidative stress. Several mechanistic studies suggested a competitive inhibition of PLA2 guided by a non-covalent molecular recognition event [103]. Because of these interesting results, a series of simplified analogues of bolinaquinone were developed, changing the sesquiterpenyl core by other rings, and the in vitro assay indicated that all compounds inhibited PGE_2_ production with different potencies [104].

Other merosesquiterpenoids with a bolinane skeleton were 21-dehydroxybolinaquinone (**247**), isolated from the Hainan sponge *D. villosa*, along with bolinaquinone (**246**) and dysidine (**249**) (Figure 22). The three compounds showed inhibitory activity of hPTP1B, a potential target for treatment of type-II diabetes and obesity [105].

Dysideamine (**248**) (Figure 22) was isolated, along with bolinaquinone (**246**), from an Indonesian sponge of *Dysidea* sp. Both compounds showed neuroprotective effects in mouse HT22 hippocampal neuronal cells, and dysideamine (**248**) inhibited the production of ROS [106].

A different rearrangement of the sesquiterpenoid skeleton led to a 6–7 carbocyclic framework as in frondosins (frondosin A, **250**) or liphagal (**251**) (Figure 22). Although these compounds were described before the period covered by this review, several synthetic studies have been performed during this time to clarify conflicting assignments on some stereocentres [107,108,109]. These were well compiled in the Wright’s review [108].

Many other cyclization patterns were described, having additional C–C bonds between the sesquiterpenic decaline and the aromatic fragments, giving rise to tetracyclic meroterpenoids after the formation of an additional cyclopentene or cyclohexene ring, as those described below.

**Figure 23:**
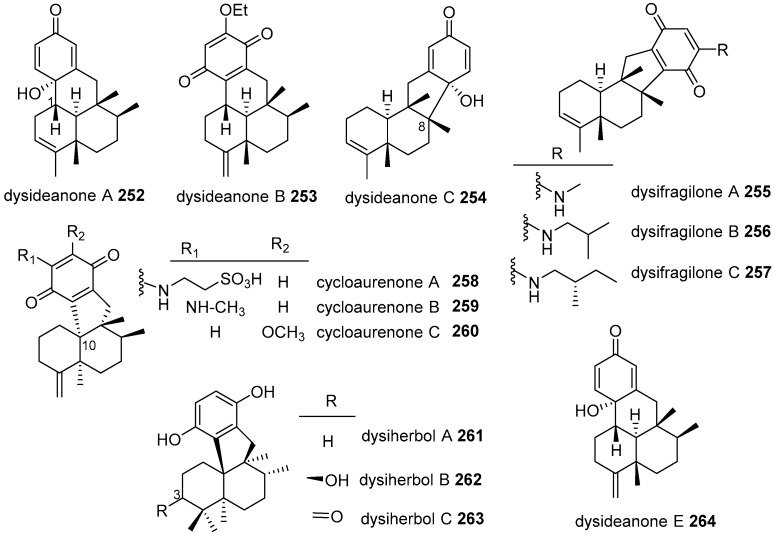
Structure of tetracyclic merosesquiterpenoids **252**–**264**.

The formation of a new C–C bond between the quinone and the C-1 position of the avarane-derived decaline generates the unprecedented 6-6-6-6-fused benzoanthracene skeleton found in dysideanones A and B (**252** and **253**) (Figure 23), isolated from the South China Sea sponge *D. avara* collected at Yongxing Island, China [110]. From the same specimen, another unusual 6-6-5-6-fused benzofluorene core was found in dysideanone C (**254**) (Figure 23), in which the additional C-C bond was formed between the quinone and the decaline C-8 position. None of the three compounds showed any antibacterial or antifungal activity, and only dysideanone B (**253**) was cytotoxic. It was postulated that avarol (**196**) (Table 5) could be their biosynthetic precursor [110].

Dysifragilones A-C (**255**–**257**) (Figure 23) are other examples of the same 6-6-5-6-benzofluorene core isolated from *Dysidea fragilis* collected at the same place, Yongxing Island, and bearing amino substituents on the quinone fragment. They displayed potential anti-inflammatory activity through the inhibition of NO production in mouse macrophages, with dysifragilone A being the most potent compound [111].

A different benzofluorene skeleton was described by the first time in cycloaurenones A–C (**258**–**260**) (Figure 23), isolated from a tropical *Dysidea* sp. collected at Chuuk Island in the Federal States of Micronesia. In these compounds, the five-membered ring was formed by a direct C-C linkage between the position C-10 of the avarane-type decaline and the quinone moiety. They exhibited cytotoxic activities comparable to that of doxorubicin. Melemeleones C and D (**171** and **172**) (Table 2) described above were also isolated from the same specimen [79].

With the same 6-6-5-6-fused tetracyclic carbon skeleton, but derived from HQs, are the dysiherbols A-C (**261**–**263**) (Figure 23), also isolated from a *Dysidea* sp. collected at Xisha Islands in the South China Sea [112]. Evaluation of their NF-kB inhibitory activity and their cytotoxicity disclosed that dysiherbol A (**261**) was several times more potent than the other two, highlighting the negative influence of oxygen functions at C-3 of the decaline. From the same specimen, dysideanone E (**264**) (Figure 23), with the same benzoanthracene as mentioned above, was also isolated [112].

**Figure 24:**
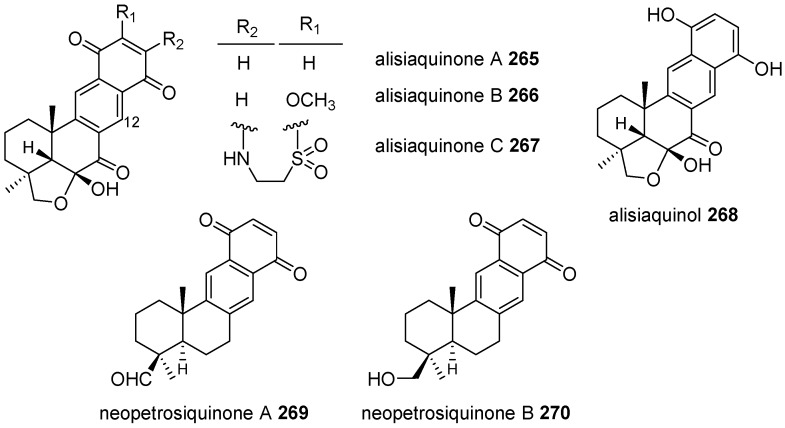
Structure of tetracyclic merosesquiterpenoids **265**–**270**.

A different 6-membered ring was formed when the new C–C bond linked the aromatic core to the C-12 methyl of a drimane decaline. This happened in alisiaquinones A–C (**265**–**267**), and in alisiaquinol (**268**) (Figure 24), which was isolated from an unidentified New Caledonian deep water sponge. The new 6-membered ring was aromatized, forming benzo[*a*]anthracenequinones. The higher oxidation degree of the decaline core allowed the formation of an additional tetrahydrofuran ring. These meroterpenoids displayed in vitro and in vivo antimalarial activity on *P. falciparum* strains, being good inhibitors of two malarial targets, the plasmodial kinase Pfnek-1 and a protein farnesyl transferase [113].

The same carbon skeleton was also present in neopetrosiquinones A and B (**269** and **270**) (Figure 24), isolated from the deep-water sponge *Neopetrosia* cf. *proxima*, collected off the north coast of Jamaica [114]. Their enantiospecific synthesis have been performed by Chayboun et al. from the labdane diterpenoid *trans*-communic acid. They showed moderate cytotoxicity against a panel of tumor cell lines and were able to induce apoptosis [115].

**Figure 25:**
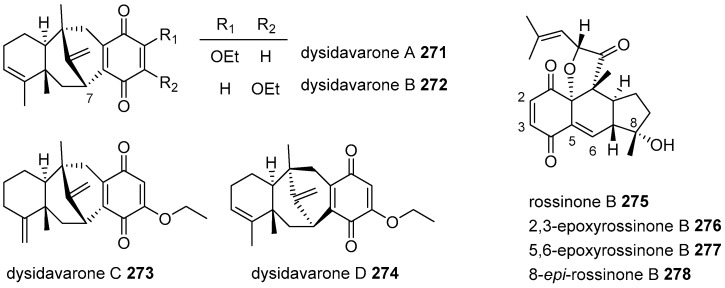
Structure of merosesquiterpenoids **271**–**278**.

A different 6-6-6-6-tetracyclic carbon skeleton, resulting from cyclization of the quinone core on the C-7 position of the avarane, was found in dysidavarones A–D (**271**–**274**) (Figure 25), and was given the name of dysidavarane. They were isolated from the sponge *D. avara*, which was collected off the Xisha Islands by Lin’s group, who proposed that they might be generated biogenetically from avarone and neoavarone. These compounds were cytotoxic for four tumor cell lines, and dysidavarones A (**271**) and D (**274**) were also inhibitors of PTP1B [48]. Because of their unusual structure, dysidavarone A (**271**) has been the objective of several total syntheses performed by different research groups [116,117,118].

There are other polycyclic meroterpenoids with rearranged skeletons, such as rossinone B (**275**) and the three NQ derivatives **276**–**278** (Figure 25), isolated from Antarctic colonial ascidians of the genus *Aplidium*, such as *Aplidium fuegiense* collected in the Western Weddell Sea, Antarctica [8,119,120]. Rossinones were allocated in the internal tissues of the ascidians, and showed significant feeding-deterrent activity against invertebrate predators and bacterial fouling, revealing their role in predation avoidance [119,120]. A biomimetic highly efficient total synthesis of rossinone B (**275**) was performed by Zhang et al. [121].

## 5. Meroterpenoids with Open-Chain and Cyclic Diterpenoids

### 5.1. Open-Chain Diterpenoids

Open-chain diterpenoids are well known and widely distributed in nature, and tocopherols or plastoquinones belong to this group of structures.

These kinds of meroditerpenoids are abundant in brown algae (Phaeophyceae). Among them, the genus *Sargassum* (Sargassaceae) has been highly studied as source of tetraprenyl-toluquinols, toluquinones, and chromenols, and they have been reported to display a range of biological activities, as described in previous reviews [1,3].

**Figure 26:**
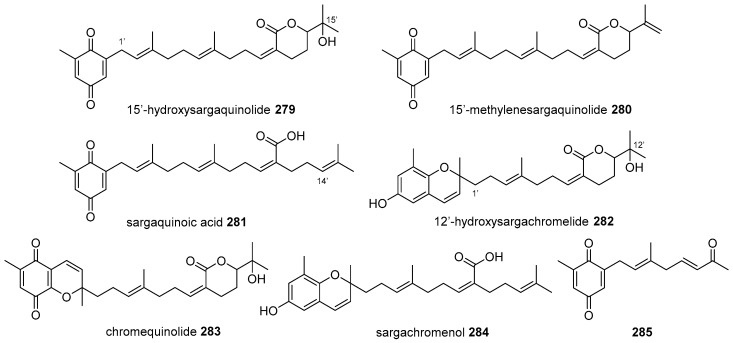
Structures of open-chain meroditerpenoids and related compound **279**–**285**.

From the species *Sargassum sagamianum*, collected at Manazuru (Japan), Horie et al. isolated two new toluquinones with δ-lactones in their tetraprenyl side chains (**279** and **280**), along with the plastoquinone sargaquinoic acid (**281**) (Figure 26), which was postulated to be epoxidized at Δ^14′^ of the side chain, followed by cyclization [122]. Moreover, in the same study, the authors obtained the chromene derivatives 12′-hydroxysargachromelide (**282**), chromequinolide (**283**), and sargachromenol (**284**) (Figure 26), which were considered as artefacts because of their lack of optical activity. One interesting ketone that was probably formed by oxidative cleavage was compound **285** (Figure 26), which showed the best results from in vitro assays as an antibacterial against *S. aureus*, and as a cytotoxic compound against HeLa S3 cells.

Sargaquinoic acid and sargachromenol displayed an apoptotic effect in vitro and in vivo, which was enhanced in combination with ultraviolet-B irradiation by caspase activation. This combination could be effective as a therapeutic agent against hyperproliferative diseases of skin, such as psoriasis [123].

**Figure 27:**
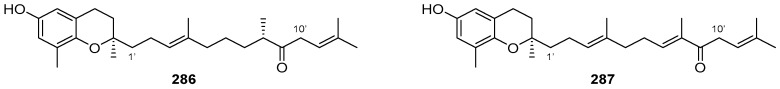
Structures of diterpenic meroterpenoids **286**–**287**.

Also from the coasts of Japan (Toyama Bay), Iwashima et al. isolated from *Sargassum micracanthum* two new chromane derivatives (**286** and **287**) (Figure 27) [124]. Their absolute stereochemistries were determined by a modified Mosher’s method through its known 10′*R*-hydroxy derivative, which was also used as semisynthetic precursor of **286** through the reduction of the 10′*R*-acetoxy derivative with SmI_2_/HOAc.

**Figure 28:**
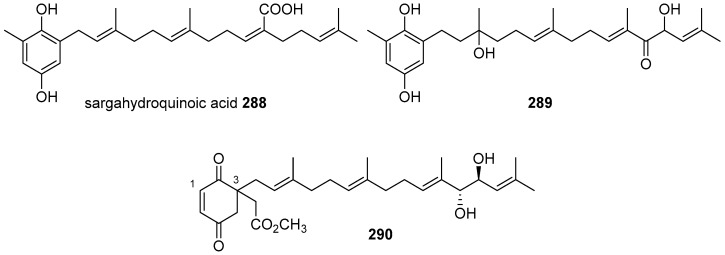
Structures of open-chain diterpenic meroterpenoids **288**–**290**.

From *Sargassum siliquastrum* collected near Jeju Island (South Korea), Jung et al. obtained the antioxidant sargahydroquinoic acid (**288**), along with the new analogue **289** with an unknown stereochemistry, and compound **290**, a derivative related to a modified tetraprenyl dihydroquinone, which is unprecedented within algal metabolites (Figure 28) [125].

**Figure 29:**
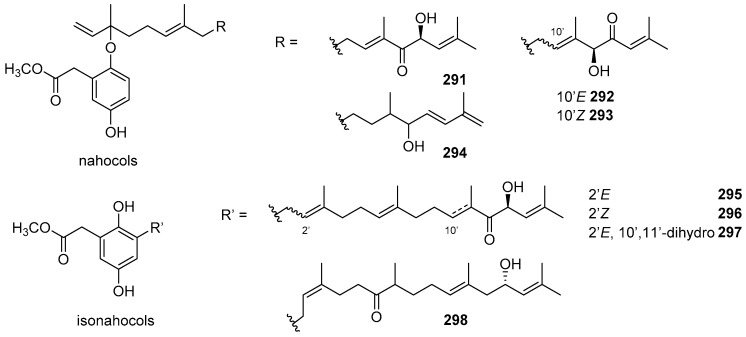
Structures of nahocol and isonahocol-related meroditerpenoids **291**–**298**.

Compound **290** could be a biosynthetic precursor of nahocols and isonahocols (Figure 29) by a 1,3-migration of its methyl acetate group. Although the biogenetic relation is unclear, this hypothesis was supported because in the same study, eight new (**291**–**298**) (Figure 29) and eight known meroditerpenoids of the nahocol and isonahocol classes were isolated. Diphenolic isonahocol compounds showed a 100-fold increase in radical-scavenging activity relative to monophenolic nahocols [125].

**Figure 30:**
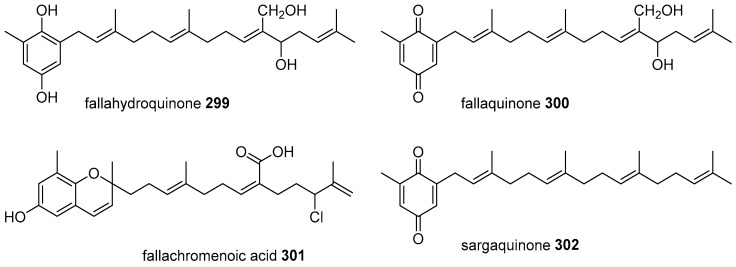
Structures of diterpenic meroterpenoids **299**–**302**.

In southern Australia (Port Phillip Bay), Reddy and Urban collected another species of this genus, *Sargassum fallax*, from which they isolated fallahydroquinone (**299**), fallaquinone (**300**, an artefact formed after exposure to air) and fallachromenoic acid (**301**), along with sargaquinone (**302**) (Figure 30), sargaquinoic acid (**281**) (Figure 26), and other known derivatives [126]. These new meroditerpenoids showed IC_50_ values around 27–29 μM against P-388 murine leukaemia cells. Their stereochemistry could not be assigned due to the rapid decomposition of the compounds isolated. In our opinion, allylic δ-chlorocarboxylic acid moieties like those present in fallachromenoic acid (**301**), could be precursors of δ-lactones related to sargachromelides (Figure 26).

**Figure 31:**
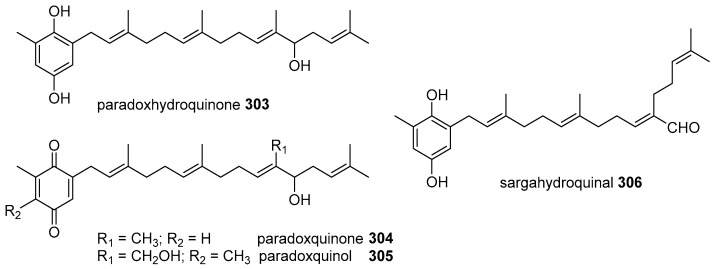
Structures of diterpenic meroterpenoids **303**–**306**.

Again Urban, together with Brkljača, described four new meroditerpenoids isolated after an antimicrobial-guided investigation from *Sargassum paradoxum* from the same sea waters, in addition to other known tetraprenyl analogues, among them, the main constituents being fallahydroquinone (**299**) (Figure 30) and sargahydroquinoic acid (**288**) (Figure 28) [127]. They used stop-flow high-performance liquid chromatography (HPLC)-nuclear magnetic resonance (NMR) and HPLC-mass spectrometry (MS) to characterize paradoxhydroquinone (**303**), paradoxquinone (**304**), paradoxquinol (**305**), and the unstable sargahydroquinal (**306**) (Figure 31).

**Figure 32:**
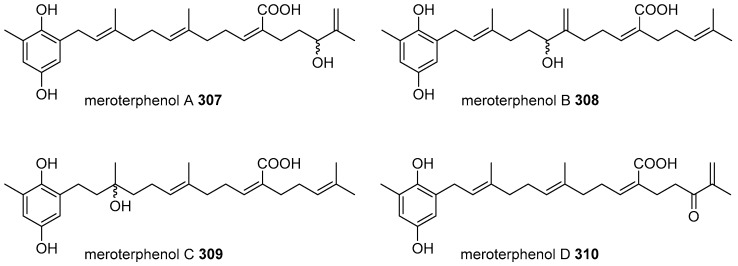
Structures of diterpenic meroterphenols **307**–**310**.

The last species of this genus reviewed in this part is *Sargassum yezoense*, which collected in the eastern coast of South Korea by Kim et al. [128]. They described four new plastoquinones designated as meroterphenols A–D (**307**–**310**) (Figure 32), which showed potent activation effects on peroxisome proliferator receptor gamma (PPARγ), so they might have anti-diabetic potential via the transactivation of this receptor.

In the same family Sargassaceae, the genus *Cystoseira* with nearly 300 species has been an important source of bioactive tetraprenyltoluquinols [129].

**Figure 33:**
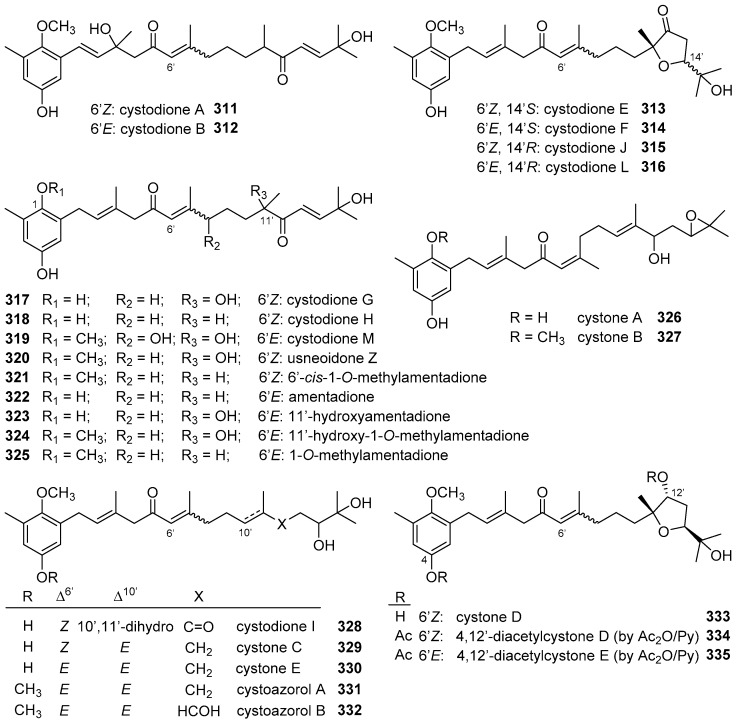
Structures of diterpenic meroterpenoids **311**–**335**.

In two different samples of *Cystoseira usneoides*, one collected at Gibraltar Strait along the Mediterranean coast of Morocco [42] and another at the coast of Tarifa (Spain) [130], de los Reyes et al. isolated 10 cystodiones (**311**–**319** and **328**) and six cystones (**326**, **327**, **329**, **330**, **333/334**, and **335**), that were new toluquinol and *O*-methyltoluquinol diterpenoids with different oxygenated functionalities and unsaturations along the diterpenoid backbone, besides other known analogues (**320**–**325**) (Figure 33). All of them showed antioxidant activity in the ABTS radical-scavenging assay. Moreover, the significant activity of **317**, **319**, **322**, **323**, and **329** as inhibitors of the production of the proinflammatory cytokine TNF-α in LPS-stimulated THP-1 macrophages (human monocytic leukaemia cells) support the potential of these products for usage as anti-inflammatories [130].

From *Cystoseira abies-marina* collected off Sao Miguel Island, Azores (Portugal), two other analogues that were completely methylated at the toluquinol motif were obtained by Gouveia et al., and were called cystoazorols A and B (**331** and **332**) (Figure 33) [41].

**Figure 34:**
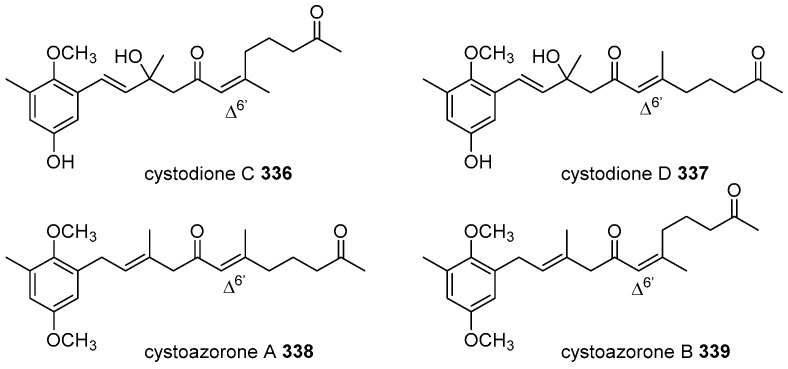
Structures of open-chain meroterpenoids **336**–**339**.

Moreover, from this species of *Cystoseira*, four new ketones with only 14 carbon atoms at the terpenyl chain were isolated. They probably derived from the oxidative degradation of the diterpenoid side chain. Cystodiones C and D (**336** and **337**) (Figure 34) differ in the configuration of Δ^6^, and can be considered to be derived from cystodiones A and B (**311** and **312**, Figure 33) [42]. Cystoazorones A and B (**338** and **339**) (Figure 34) are also Δ^6^ stereoisomers, and they showed moderate antioxidant and anti-inflammatory activities [41].

**Figure 35:**
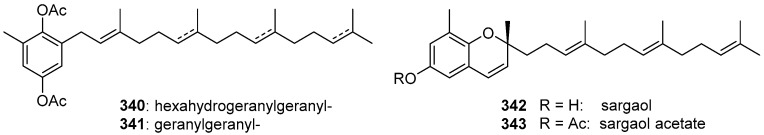
Structures of diterpenic meroterpenoids **340**–**343** from *S. flabelliforme.*

Another marine brown algae, *Stypopodium flabelliforme* (Dictyotaceae) was collected in Easter Island (Chile) by Areche et al., and after acetylation of the extract, tetraprenylhydroquinone diacetate **340** (Figure 35) was isolated [131]. It was described by the authors as a new natural product, although it was synthesized many years ago in investigations on the biosynthesis of α-tocopherols in spinach chloroplasts [132]. From the same peracetylated extract, the known diacetylated geranylgeranyl analogue **341** and its acetylchromenol derivative **343** were isolated (Figure 35), along with some tricyclic meroditerpenoids, which are presented later in Section 5.4. Six years later, the same group published the gastroprotective effects of sargaol (**342**) (Figure 35) in a mouse model, which was attenuated with indomethacin and *N*-ethylmaleimide, suggesting that prostaglandins and sulfhydryl groups are involved in its mode of action. Sargaol (**342**) was obtained after hydrolysis of the acetylated derivative **343** [133].

**Figure 36:**
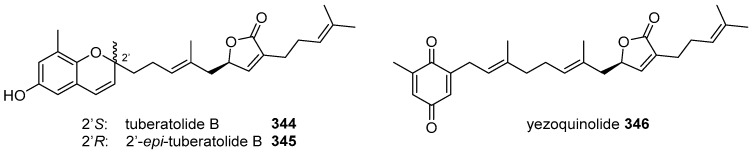
Structures of diterpenic meroterpenoids **344**–**346** from *B. tuberatus.*

Diterpenylquinones and derivatives were also isolated from other marine organisms different from brown algae, such as ascidians, sponges or soft corals, all belonging to the animal kingdom. From the ascidian *Botryllus tuberatus*, two new epimeric γ-lactones, tuberatolide B (**344**) and 2′-*epi*-tuberatolide B (**345**) (Figure 36) were isolated [134]. The co-isolation in equivalent amounts of both chromenols suggests that they could have originated as artefacts from the known quinone yezoquinolide (**346**) (Figure 36), which was also isolated from this Korean marine tunicate, along with the two separated enantiomers of sargachromenol (**284**) (Figure 26). As these last compounds had already been described in different brown algae of genus *Sargassum* [135,136], it would be possible that symbiotic microorganisms in brown algae and tunicates synthesized these terpenoids or alternatively, that the source organisms may have similar biosynthetic genes. These tuberatolides were potent (1.5–2.5 μM) antagonists of chenodeoxycholic acid-activated human farnesoid X receptor (hFXR), and this fact may help to unravel the controversial function of FXR in atherosclerosis.

**Figure 37:**
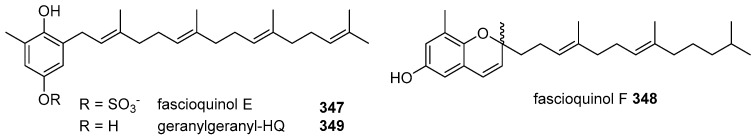
Structures of diterpenic meroterpenoids **347**–**349** from *Fasciospongia* sp.

During a big program for discovering new anticancer agents, the group of Capon described the new meroterpenoids fascioquinol E (**347**) and fascioquinol F (**348**) from an Australian sponge, *Fasciospongia* sp., along with the known geranylgeranyl-HQ **349** (Figure 37), which was the dominant cytotoxic compound of the ethanolic extract of this sponge [137]. They proposed both tetraprenyl-HQs as the acid-mediated biosynthetic precursors of the tricyclic fascioquinols, which are described later.

**Figure 38:**
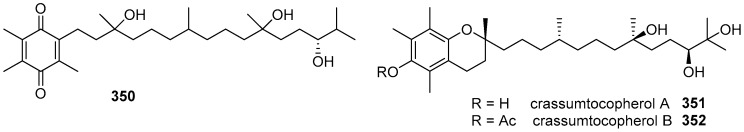
Structures of diterpenic meroterpenoids **350**–**352** from soft corals.

From a soft coral collected off the South China Sea, *Sarcophyton infundibuliforme*, Wang et al. isolated α-tocopherylquinone **350** (Figure 38), which was quite rare in marine materials [138]. Also, from another soft coral, the Formosan *Lobophytum crassum*, crassumtocopherols A (**351**) and B (**352**) (Figure 38) were obtained, these two new α-tocopherols having moderate cytotoxicity against P-388 cells [139].

### 5.2. Monocyclic Diterpenoids

**Figure 39:**
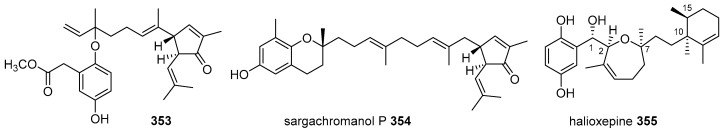
Structures of diterpenic meroterpenoids **353**–**355**.

In addition to the open-chain meroditerpenoids of the nahocol class from *S. siliquastrum* mentioned above [125], isolation was made for compound **353** (Figure 39), which had a cyclopentenone tetraprenyl chain. Only one natural compound has previously shown this cyclization pattern in brown algae, sargachromanol P (**354**) (Figure 39), a chromane meroterpenoid that was isolated from the same species [140].

A new carbon skeleton was found for halioxepine (**355**) (Figure 39), a HQ with a diterpenic motive containing a cyclohexene and another additional oxepin ring, isolated from *Haliclona* sp., an Indonesian sponge. It showed moderate cytotoxicity against NBT-T2 cells (rat bladder epithelial carcinoma) [141]. Only the relative configuration of the stereocenters in each ring, 1, 2 and 7, and 10 and 15, was assigned.

**Figure 40:**
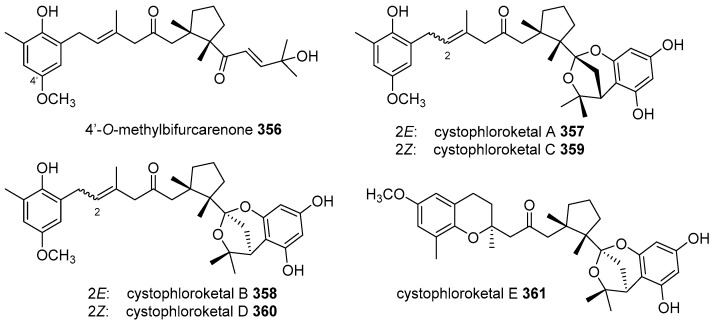
Structures of diterpenic meroterpenoids **356**–**361** from *C. tamariscifolia.*

From the brown alga *Cystoseira tamariscifolia* collected off the Algerian coasts, the known monocyclic meroditerpene 4′-*O*-methylbifurcarenone (**356**) (Figure 40) was isolated. It was considered to be the precursor of the also isolated cystophloroketals A–E (**357**–**361**) (Figure 40), which showed antifouling activity [142]. These new compounds are hybrid natural products with three distinct units: a diterpene linked to a *O*-methyltoluquinol and a phloroglucinol. Although terpenyl-phloroglucinols are well known [143], these hybrids are the first examples of meroterpenoids with a 2,7-dioxabicyclo[3.2.1]octane backbone fused to a phloroglucinol.

### 5.3. Bicyclic Diterpenoids

**Figure 41:**
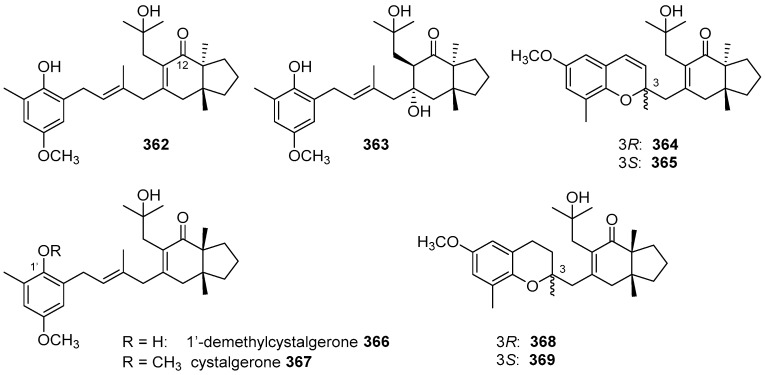
Structures of diterpenic meroterpenoids **362**–**369** from *C. baccata.*

Four new bicyclic diterpenoids linked to a HQ derivative (**362**–**365**) (Figure 41) were isolated by Mokrini et al., from *Cystoseira baccata* harvested along the Atlantic coasts of Morocco [144]. The absolute configuration was deduced by application of modified Mosher’s method through the hydroxy derivative at C-12. The stereostructure of the hydrindane system of these compounds revealed a *trans* orientation of their two bridgehead methyls, instead of the *cis* orientation of compounds previously reported from *Cystoseira* genus as 1′-demethylcystalgerone (**366**) and its chromanol derivatives (**368** and **369**) (Figure 41) [145,146]. On the basis of the NMR data and optical activity comparisons, Mokrini et al. [144] postulated the hypothesis that the compounds related to cystalgerone (**367**) were isolated at least from the species *C. baccata* [147,148], would possess a *trans*-fused configuration.

**Figure 42:**
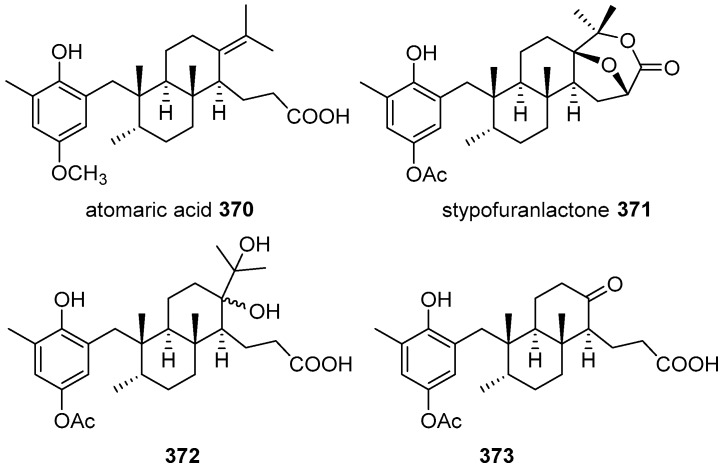
Structures of bicyclic diterpenic meroterpenoids **370**–**373**.

In a study on intraspecific variation of meroditerpenoid production carried out at four different populations of Brazilian brown alga *Stypopodium zonale*, three chemotypes were described through their HPLC-DAD chemical profiles of their CH_2_Cl_2_ extracts. Along with the principal and known bicyclic meroditerpene atomaric acid (**370**) and the tricyclic stypoldione, three new carbobicyclic compounds (**371**–**373**) (Figure 42) were isolated and fully characterized by NMR and MS techniques [149]. It is the first time that a compound with the furan-lactone moiety of stypofuranlactone (**371**) has been obtained from marine brown algae.

### 5.4. Tricyclic and Tetracyclic Diterpenoids

**Figure 43:**
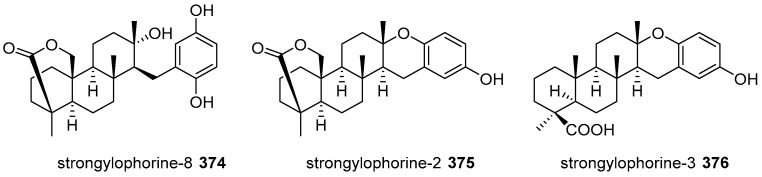
Structures of strongylophorines **374**–**376**.

The strongylophorines are meroditerpenoids that were isolated by Braekman et al. for the first time from the sponge *Strongylophora durissima*, actually belonging to genus *Petrosia* and subgenus *Strongylophora* [150]. Several activities have been described in the last years for them, as antibacterial, antifungal, insecticidal or cytotoxic against different cancer cells, among others. From *Petrosia (Strongylophora) strongylata* collected off Milne Bay (Papua New Guinea), the known strongylophorines-8, -2, and -3 (**374**–**376**) were isolated (Figure 43). They were responsible for the inhibition of the HIF-1, considered as a crucial mediator in survival and progression of tumors [151]. The tricyclic diterpenoid, including the lactone fragment present in **374** and **375**, was important for HIF-1 inhibition.

**Figure 44:**
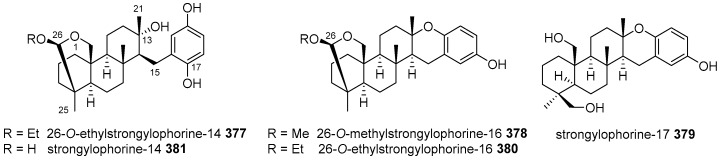
Structures of diterpenic meroterpenoids **377**–**381**.

In the same species collected off Okinawa (Japan), the new 26-*O*-ethylstrongylophorine-14 (**377**) (Figure 44) was isolated by Lee et al., along with several known compounds of this family, some of them represented here (**374**–**376**, **378**, **379**) (Figure 43 and Figure 44) [152]. Their activity as PTP1B inhibitors was evaluated, being stronger for compounds that had an acetal moiety at C-26 than for those with a lactone function.

From the Indonesian sponge *Petrosia corticata*, 26-*O*-methylstrongylophorine-16 (**378**) and 26-*O*-ethylstrongylophorine-16 (**380**) were isolated (Figure 44), along with the known derivatives **374**–**376** and **381**. Their inhibitory activity of the proteasome was tested, the acetals being the most active compounds **377**, **378**, **380**, and **381**. Additionally, the HQs were more active than chromanols, as happened with strongylophorine-14 (**381**) and its epimer at C-26, the hemiacetal position [153].

**Figure 45:**
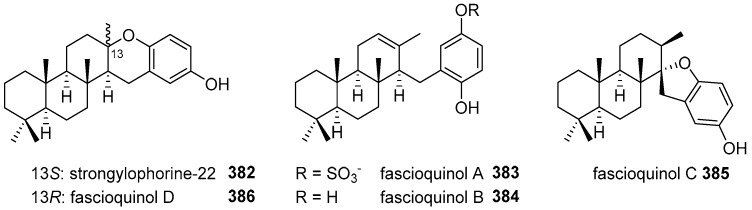
Structures of diterpenic meroterpenoids **382**–**386**.

Beside the geranylgeranyl-HQ **349** and fascioquinols E and F (**347** and **348**) (Figure 37) mentioned previously, from an Australian marine sponge, *Fasciospongia* sp., several tricyclic meroditerpenoids, as the known strongylophorine-22 (**382**) and the new meroterpene sulfate fascioquinol A (**383**), were isolated (Figure 45). A series of acid-mediated hydrolysis/cyclization products, fascioquinols B–D (**384**–**386**) (Figure 45), were also isolated, and they were biosynthetically related to the open-chain diterpenoids. Fascioquinols A and B (**383** and **384**) showed promising selective Gram-positive antibacterial activity towards *S. aureus* and *B. subtilis* [137].

**Figure 46:**
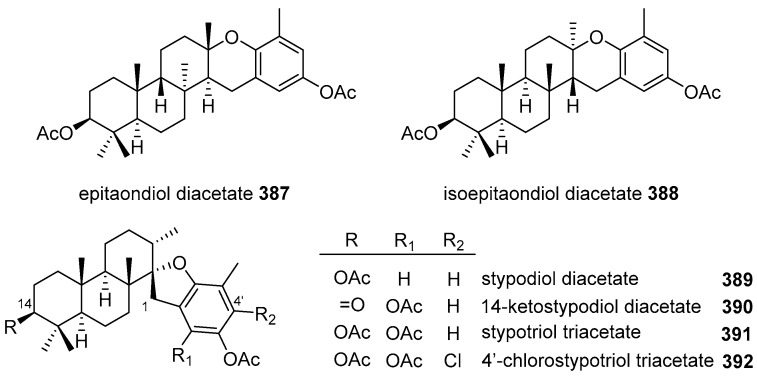
Structures of diterpenic meroterpenoids **387**–**392**.

The simultaneous presence of open-chain and tricyclic meroditerpenoids in the same organism was also observed in *S. flabelliforme*, as mentioned before, and this could support the biogenetic relationship between them. Meroterpenoids are usually isolated as their corresponding acetates, to avoid the over-oxidation of the natural products. Thus, in addition to the acetylated meroterpenoids **340**–**343** described above (Figure 35), the known tricyclic epitaondiol, isoepitaondiol, stypodiol, and 14-ketostypodiol diacetates (**387**–**390**), stypotriol triacetate (**391**), and the new 4′-chlorostypotriol triacetate (**392**) (Figure 46) were isolated after acetylation of the extract [131]. The occurrence of halogenated compounds is unusual in brown seaweed, and **392** was the first halocompound reported in the *Stypopodium* genus.

**Figure 47:**
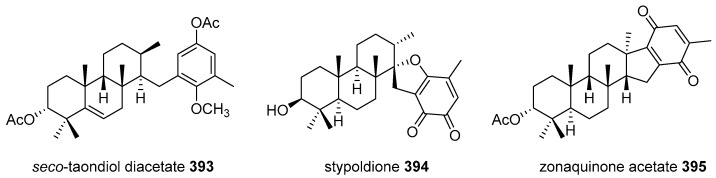
Structures of diterpenic meroterpenoids **393**–**395**.

The acetylated derivative of *seco*-taondiol (**393**) (Figure 47), a new meroterpenoid with an unprecedented in nature *syn-cis-anti* arrangement for the A/B/C ring system, was isolated from *S. flabelliforme*, along with 10 known meroterpenoids, and it was evaluated as being gastroprotective; after hydrolysis of the acetates, it was the most active epitaondiol, isoepitaondiol, stypodiol (Figure 46) and sargaol (**342**) (Figure 35) through different modes of action [133].

From *S. zonale* collected off distinct areas of the Brazilian coast, besides the bicyclic meroterpenoids **370**–**373** mentioned before (Figure 42), the known tricyclic stypoldione (**394**) (Figure 47) was also isolated [149]. From the same *S. zonale* but collected off Jamaica, Penicooke et al. described the only tetracyclic derivative reported in this period, zonaquinone acetate (**395**), along with stypoldione (**394**) (Figure 47) and sargaol (**342**) (Figure 35). The tetracyclic diterpenoid was fused to a methylBQ and the acetate was naturally present in the extract. It was assayed favorably as being antiproliferative against MCF-7 breast cancer and HT-29 colon cancer cells [154].

## 6. Meroterpenoids with Larger Terpenoids

**Figure 48:**
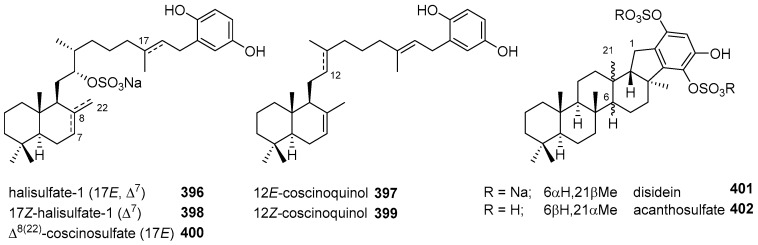
Structures of sesterterpenic meroterpenoids **396**–**402**.

Only three new sesterterpenoid HQs were discovered over these years, with two of them being related to the bicyclic terpenoid halisulfate-1 (**396**) and the other to disidein (**401**), the latter presenting a pentacyclic system in the terpene core (Figure 48).

Two new pentaprenyl bicyclic HQs, 12*E*-coscinoquinol (**397**) and 17*Z*-halisulfate-1 (**398**), and the other three known compounds, halisulfate-1 itself (**396**), 12*Z*-coscinoquinol (**399**), and the sodium salt of the coscinosulfate isomer **400**, were obtained from a Micronesian (Chuuk Island) sponge, *Coscinoderma* sp. (Figure 48) [155]. The sulfate-containing compounds were more potent than the coscinoquinols as Na^+^/K^+^-ATPase inhibitors, and against sortase A, a key enzyme for the cell adhesion of Gram-positive bacteria. However, coscinoquinols showed more potent inhibition than doxorubicin against K562 cells, while the halisulfates were inactive.

With scalarene skeleton, acanthosulfate (**402**) was described as a new derivative of disidein (**401**) (Figure 48), with different configuration at several stereocenters [156]. It was isolated by West and Faulkner from a Philippine sponge, *Acanthodendrilla* sp., collected off Boracay Island. Acanthosulfate inhibited the proteasome function (IC_50_ 4.5 μM); however, it lacked the selectivity and potency in the BMS Oncology Diverse Cell Panel (ODCA) to be considered for in vivo evaluation.

**Figure 49:**
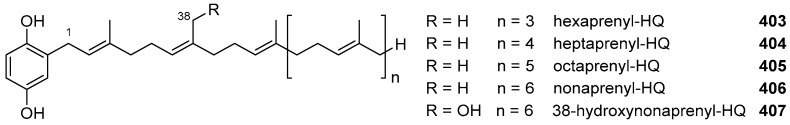
Structures of polyprenylated HQs **403**–**407**.

Finally, from different Mediterranean sponges, several all-*trans* polyprenylated HQs were isolated: hexaprenyl-HQ (**403**) and nonaprenyl-HQ (**406**) from *Sarcotragus muscarum* [157], heptaprenyl-HQ (**404**) from *Ircinia fasciculata* [157], and again **404**, octaprenyl-HQ (**405**), and the new hydroxylated at C-38 nonaprenyl-HQ **407** from *Sarcotragus spinosulus* [158] (Figure 49). Hexaprenyl-HQ (**403**) showed a great cytotoxicity against H4IIE hepatoma cells and also resulted in potent inhibitors of EGFR tyrosine kinase, among other kinases [157].

## 7. Preparation of Bioactive TQs/HQs from Inactive Terpenoids

As has been seen along this review, TQs/THQs are secondary metabolites with very interesting and sometimes unusual structural features, and also with a great variety of biological activities (anti-inflammatory, cytotoxic, antibacterial, antifungal, etc.), very often with difficult evaluation, due to the small amounts obtained from their natural sources, and even with difficulties for collecting again from the same organism. These facts have attracted scientific attention, and many efforts have been devoted to their synthesis, not only for confirming both the proposed structures and their absolute configurations, but also to obtain enough amounts to perform additional biological evaluations.

Some of this kind of work has already been mentioned within this review, particularly that which deals with the total synthesis of the new TQs/THQs described here. Sometimes, the synthesis started from easily available small molecules such as the (+)-5-methyl Wieland-Miescher ketone used by Katoh et al. for the synthesis of several sesquiterpene quinones [159], or the Marshall’s decaline used by Kobayashi et al. in the synthesis of the akaterpin [160]. In other cases, natural products were used as the starting material. The strategies used in the synthesis of different TQs/THQs were the main subject of previous reviews [108,161], so this section is not intended to be exhaustive, but rather to focus in the idea of using natural products, particularly terpenoids, as the starting point to obtain a variety of bioactive analogues of marine natural products.

**Figure 50:**
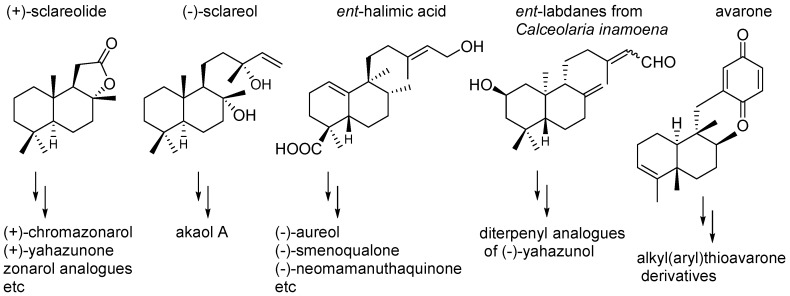
Structures of natural compounds used as starting material for the synthesis of several TQs.

A number of natural terpenoids obtained from their natural sources in enough quantity or industrial by-products with decaline cores, such as those that are present in the sesquiterpenequinones described here, can be used to synthesize many meroterpenoids. Using natural terpenoids has the advantage of starting from enantiopure compounds, so that single enantiomers can be obtained. Among those are (+)-sclareolide (Figure 50), a common feedstock in the perfume industry used by Baran et al. for the synthesis of 10 meroterpenoids with drimane skeletons [162]. Álvarez-Manzaneda et al. used (−)-sclareol, from *Salvia sclarea* (Fam. Lamiaceae), for the synthesis of akaol A [163]. Several *ent*-labdanes isolated from *Calceolaria inamoena* (Fam. Scrophulariaceae) were used by Catalan et al. for the synthesis of drimane-quinones [164], and *ent*-halimic acid, isolated from *Halimium viscosum* (Fam. Cistaceae), was used by Marcos et al. for the synthesis of several TQs with aureane skeleton (Figure 50) [165].

Natural terpenoids can also be used to obtain families of analogues that would allow for the performance not only different biological evaluations but also to analyze structure-activity relationships to establish the main structural features for a defined bioactivity, in order to design better drug candidates.

Avarone (**139**) (Table 1) itself, which can be isolated in big quantities from *D. avara*, was used to obtain new non-natural alkyl(aryl)thio derivatives by nucleophilic addition of thiols or thiophenols to the quinone. It was found that they had similar cytotoxic activity than avarone but none of them showed better antibacterial activity than avarone (Figure 50). The cytotoxicity was enhanced by electron-donating substituents that lowered the one-electron reduction potential; however, the disubstitution led to a significant decrease in potency [166].

**Scheme 1:**
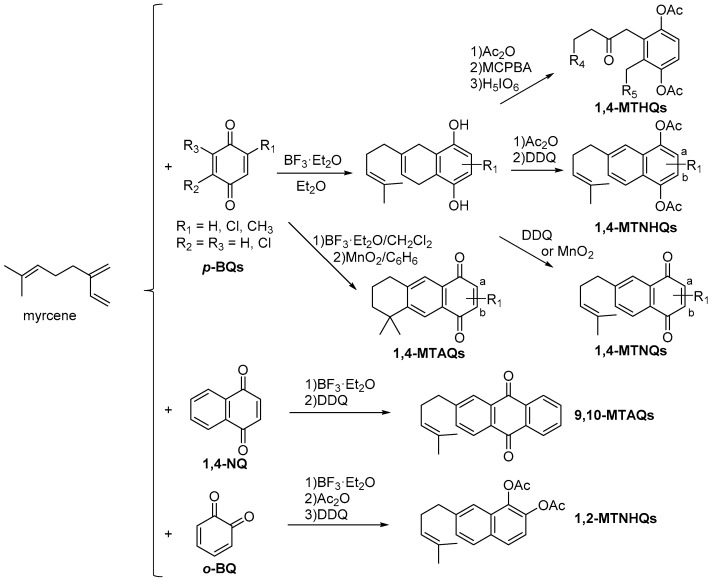
Preparation of several types of monoterpenylquinones/HQ (MTQ/MTHQ) derivatives from myrcene.

**Scheme 2:**
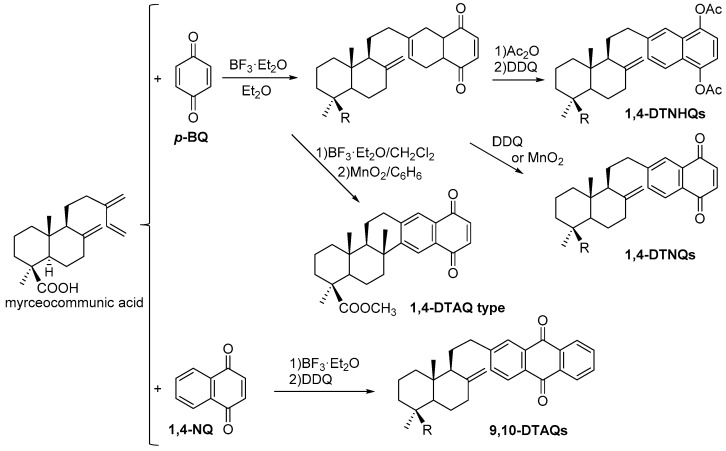
Preparation of several types of diterpenylquinones/HQs (DTQ/DTHQ) derivatives from myrceocommunic acid.

The structure of avarol (**196**) (Table 5) and avarone (**139**) (Table 1) inspired the research line developed at our laboratory for several years towards the chemo-induction of bioactivity on inactive abundant terpenoids with decalines that resembled those of the bioactive marine sesquiterpenylquinones. Thus, we designed and prepared several series of TQs/THQs, with variations in sizes and functionalities of both the quinone, BQ, NQ, or anthracenequinone (AQ), and the terpenoid (mono- or diterpene) moieties. As starting terpenoids, the monoterpene myrcene and the diterpenoid myrceocommunic acid were chosen, giving rise to two series of derivatives: monoterpenylquinones/HQs (MTQs/MTHQs) and diterpenylquinones/HQs (DTQs/DTHQs) as depicted in Scheme 1 and Scheme 2. Myrcene is commercially available, and myrceocommunic acid can be easily isolated in enough amount from the berries of *Juniperus oxycedrus* (Fam. Cupressaceae) [167].

Both terpenoids bear a conjugated diene that can serve as diene in Diels-Alder cycloadditions, the synthetic procedure used for the preparation of both series of meroterpenoids, using differently substituted *p*-BQs as dienophiles.

The cycloadducts, either from myrcene (Scheme 1) or from myrceocommunic acid (Scheme 2), were stabilized as their acetates followed by aromatization yielding acetylated naphthohydroquinone (NHQ) derivatives [167,168]. Direct oxidation of the cycloadducts led to the corresponding 1,4-NQ derivatives. When monosubstituted *p*-BQs were used, mixtures of regioisomers were obtained in different ratios [169,170]. In addition, the change of the cycloaddition solvent from ethyl ether to dichloromethane, led to the corresponding 1,4-AQ derivatives as the result of the cycloalkylation of the naphthalene core by the prenyl chain. This cyclization was due to the presence of acid traces in the reaction, as shown by several mechanistic studies performed by us [171,172].

The size of the quinone was enlarged towards 9,10-AQs when 1,4-NQ was used as a dienophile [167,168] and it was also shortened to HQ derivatives by oxidative cleavage of the trisubstituted double bond in the dihydronaphthalene ring of the cycloadduct [173,174].

Also 1,2-NHQ derivatives were similarly obtained when *o*-BQ was used in the Diels-Alder cycloaddition [175,176].

These synthesized quinone/HQ derivatives were evaluated as cytotoxic against several tumoral cell lines. All of them exhibited IC_50_ values at the micromolar level or below, improving the cytotoxicity of the unsubstituted 1,4-NQ significantly, as the 1,4-NQ analogues were more potent than both the corresponding 9,10-AQs and BQs, thus showing the importance of both nature and size of the quinone/HQ systems and the presence of substituents on the quinone/HQ core. There were no significant differences between the quinones and the corresponding acetylated HQs, and those derived from the diterpenoid were slightly more potent that those derived from myrcene.

**Scheme 3:**
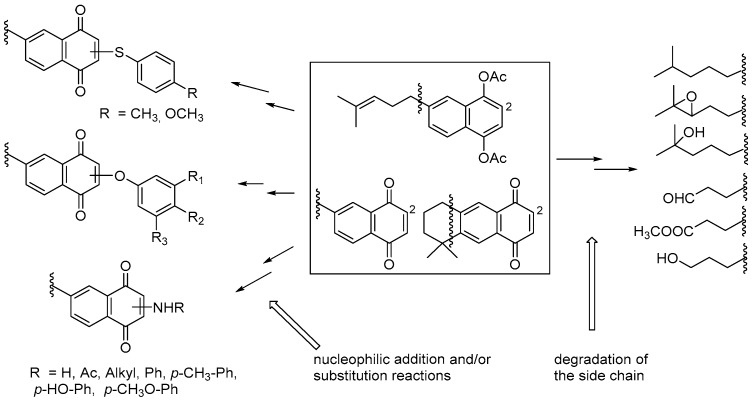
Transformations performed on the quinone ring and on the terpenoid rest in MTQs/MTHQs.

**Scheme 4:**
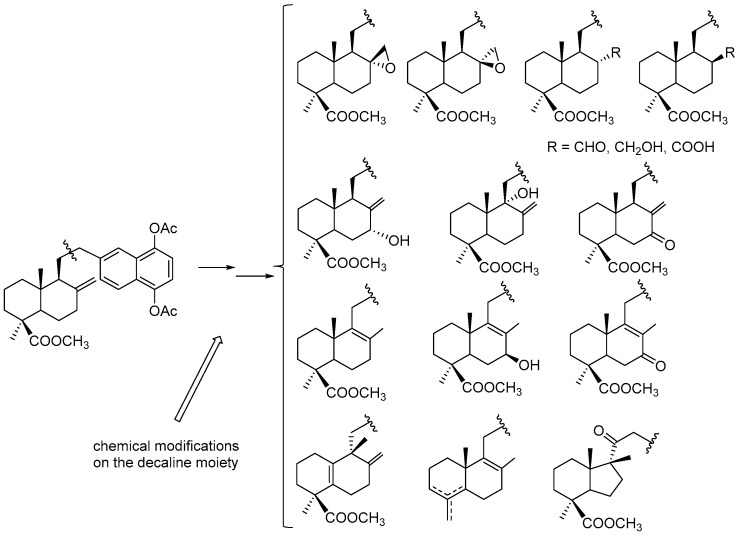
Transformations performed on the terpenic moiety of DTQs/DTHQs.

Many derivatives were obtained by chemical transformations performed either on the quinone ring or in the terpenoid fragment. For the terpenoid modifications, simple chemical transformations as hydrogenation, epoxidation and oxidative cleavage, isomerization, allylic reactions, rearrangements, etc. of the double bonds were performed on both series of MTQs/MTHQs (Scheme 3) [175,177,178] and DTQs/DTHQs (Scheme 4) [167,179]. No improvement of the cytotoxicity was observed for MTQs/MTHQs, but better results were found for DTQs/DTHQs. Changes on the quinone system included nucleophilic additions and substitution reactions using sulphur, nitrogen, and oxygen nucleophiles [170] (Scheme 3). The biological assays showed an improvement of cytotoxicity for those substituted at C-2, particularly for alkyl- or aryl-amino groups without differences between regioisomers.

**Figure 51:**
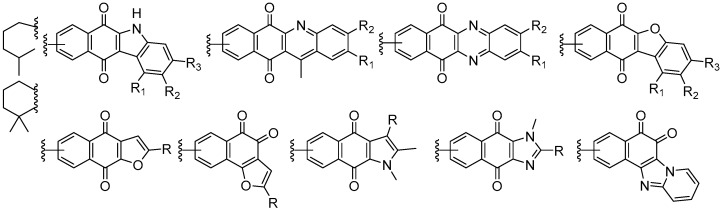
Structure of heterocyclic-fused MTQs.

Enlargement of the aromatic polycyclic system with fused-heterocycles, such as benzocarbazole, benzacridine, or the benzofuran system among others, led to less potent analogues [180]. In addition, condensation reactions of MTQs with aldehydes led to 1,4- and 1,2-furoquinones, whose biological evaluation showed that 1,2-furoquinones were better cytotoxic than the corresponding 1,4-furoquinones [181]. Other heterocyclic derivatives included pyrrol and imidazole-fused rings [182] (Figure 51).

In addition to the antineoplastic cytotoxicity assays, several MTQs/MTHQs, either derived from 1,4-NQs or 1,4-AQs, were evaluated as antifungal [182,183] and antiviral [182], with the finding that several heterocyclic derivatives showed antifungal MIC values in the low μM range against yeasts and filamentous fungi, and discrete anti-herpetic activity. The most recent and promising results include the evaluation of their anti-leishmanial activity [unpublished results].

**Figure 52:**
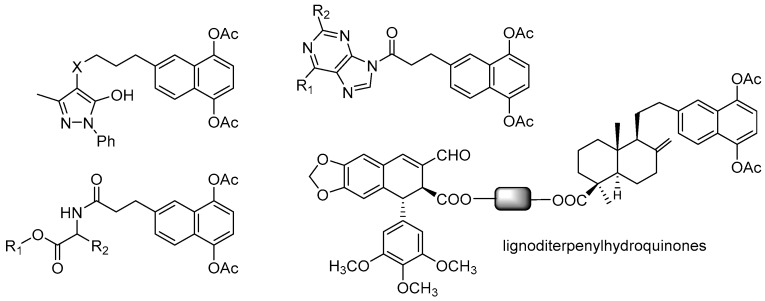
Several hybrid derivatives of MTHQs and DTHQs.

Considering the potentiality of dual targeting in drug design and development, several quinone/HQ hybrids with nitrogen heterocycles [184,185] and with other biogenic compounds as aminoacids, purines [186] and cyclolignans [187] (Figure 52), were obtained and bioassayed. The latter were named as lignoditerpenylhydroquinones, and they showed a very interesting and selective cytotoxicity against the osteosarcoma cell line MG-63, which is a very aggressive cancer, opening a new perspective for its treatment [187].

## 8. Concluding Remarks and Perspectives

The sea is an outstanding source of resources, many of them still unknown and mostly unexplored. It is worth noting the diversity of novel skeletons and structures of secondary metabolites from marine macro- and micro-organisms, often incremented by their symbiosis. Many of these metabolites have a wide variety of biological properties, so they may be susceptible to direct therapeutic use, or they may be used as models to obtain improved analogues and derivatives.

In this review, marine secondary metabolites from mixed biogenetic origins that contain a quinone/HQ moiety linked to terpenoids are described, considering their marine sources and their biological properties. They have been isolated from a variety or marine organisms, mainly from sponges, ascidians, and marine-derived microorganisms, but also there are many examples of compounds isolated from other organisms, such as algae or soft corals, most of them collected at Mediterranean, Caribbean, Okinawan, Australian, and South China Seas.

The found terpenoid fragments range from the simple isoprenyl chain, to monoterpenyl, sesquiterpenyl, diterpenyl, and larger terpenyls. The largest group is the sesquiterpenyl derivatives that can bear acyclic, monocyclic, or bicyclic sesquiterpenoid side chains. The bicyclic ones are mainly derived from drimane, aureane, and avarane skeletons. The quinonic part is usually a BQ/HQ moiety, and the corresponding cyclized chromanols, being NQ derivatives, are less frequent.

In these kinds of mixed biogenetic compounds, the hydrophilic quinone/HQ part can participate in reversible and irreversible metabolic processes of oxidation, reduction, alkylation, etc., which are fundamental for good cellular development and survival. It can also interact reversibly with other biological targets by activating or antagonizing their responses and effects. Regarding the attached terpenic part, essentially lipophilic, it can facilitate interactions with membranes and other lipidic regions, and modulate the pharmacokinetics of potential quinonic drugs, and it can even interact with other biotargets, depending on its degree of functionality and its polarity. This could explain the diverse biological activities that have been described for TQs/THQs such as antitumor, antibacterial, antifungal, antiplasmodial, anti-inflammatory, and anti-oxidant, among others, sometimes with very promising results. Although it is difficult to compare the results of cytotoxicity assays performed with different protocols, in general, those found for sesquiterpenyl derivatives are usually more potent than those for compounds with smaller or larger prenyl fragments.

In summary, marine organisms still are a good source of new bioactive compounds that are related to TQs; however, the small amount that is usually isolated limits considerably their potential development as drug candidates. Thus, much research is currently devoted to developing good synthetic procedures that allow larger quantities to be used for in vivo assays. The efforts should not only be directed towards the natural compounds themselves, but also to their derivatives and analogues, in order to obtain compounds that are more potent and selective than the original natural product. In this sense, very good results have been obtained from accessible inactive diterpenoids used as starting compounds in the synthesis towards new bioactive quinonic meroterpenoids.

Marine chemistry that is devoted to the isolation, structure assignment, transformation, and synthesis of old and new compounds and their analogues, is currently well-established in outstanding institutions of many countries, which are continuing the research on developing new drugs of marine origin. Thus, to attain better profits from these resources, further biochemical-biological-pharmacological assays focused on unresolved diseases, like central nervous system-degenerative and orphan diseases, should be systematically implemented and applied to evaluate most marine products. Similarly, the virtual computerized screening for prediction and quantification of activity, toxicity, secondary effects and pharmacokinetics of compounds with novel structures and skeletons, should take its part in marine compounds research, because today it constitutes a very useful tool for the discovery and optimization of candidates to preclinical evaluation and development of better drugs.

## Figures and Tables

**Table 1:** Meroterpenoid analogues of avarone with a *trans*-decaline.

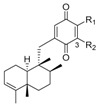				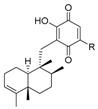		
**R_1_**	**R_2_**	**Name**		**R**	**Name**	
H	H	avarone	**139**	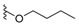	nakijiquinone S	**148**
H	NH_2_	3′-aminoavarone	**140**	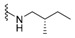	nakijiquinone K	**149**
H	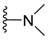	dysidaminone C	**141**	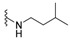	nakijiquinone N	**150**
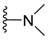	H	dysidaminone D	**142**	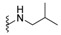	nakijiquinone O	**151**
H	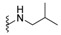	dysidaminone E	**143**	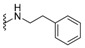	nakijiquinone Q	**152**
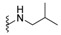	H	dysidaminone A	**144**	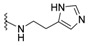	nakijiquinone G	**153**
H	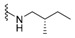	dysidaminone B	**145**	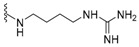	nakijiquinone H	**154**
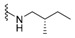	H	dysidaminone F	**146**	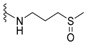	nakijiquinone I	**155**
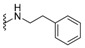	H	dysidaminone G	**147**	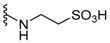	nakijiquinone R	**156**

**Table 2:** Meroterpenoid analogues of neoavarone with a *trans*- or *cis*-decaline.

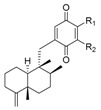				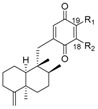			
**R_1_**	**R_2_**	**Name**		**R_1_**	**R_2_**	**Name**	
H	H	neoavarone	**157**	H	H	arenarone	**166**
H	OEt	18-ethoxyneoavarone	**158**	H	NH_2_	18-aminoarenarone	**167**
OEt	H	19-ethoxyneoavarone	**159**	NH_2_	H	19-aminoarenarone	**168**
H	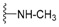	dysidaminone H	**160**	H	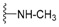	18-methylaminoarenarone	**169**
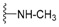	H	dysidaminone I	**161**	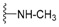	H	19-methylaminoarenarone	**170**
H	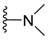	dysidaminone J	**162**	H	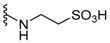	melemeleone C	**171**
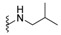	H	dysidaminone K	**163**	H	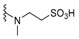	melemeleone D	**172**
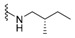	H	dysidaminone L	**164**				
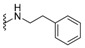	H	dysidaminone M	**165**				

**Table 3:** Meroterpenoid analogues of neoavarone with a *trans*-decaline.

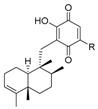					
**R**	**Name**		**R**	**Name**	
OEt	ethylsmenoquinone	**173**	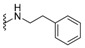	smenospongidine (smenospongiadine)	**180**
OCH_3_	ilimaquinone	**174**	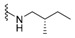	nakijiquinone L	**181**
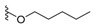	langcoquinone C	**175**	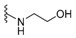	langcoquinone F	**182**
NH_2_	smenospongine	**176**	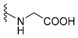	smenospongine B (glycinylilimaquinone)	**183**
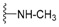	smenospongimine (langcoquinone A)	**177**	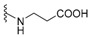	smenospongine C	**184**
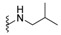	smenospongorine	**178**	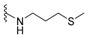	langcoquinone B	**185**
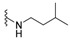	smenospongiarine	**179**	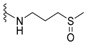	langcoquinone D	**186**
			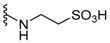	langcoquinone E	**187**

**Table 4:** Meroterpenoid analogues of avarone or neoavarone with a *cis*-decaline.

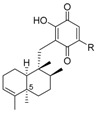			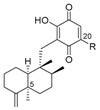		
**R**	**Name**		**R**	**Name**	
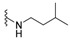	5-*epi*-nakijiquinone N	**188**	OEt	5-*epi*-20-*O*-ethylsmenoquinone	**193**
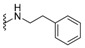	5-*epi*-nakijiquinone Q	**189**	NH_2_	5-*epi*-smenospongine	**194**
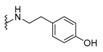	5-*epi*-nakijiquinone S	**190**	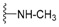	*N*-methyl-5-*epi*-smenospongine	**195**
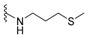	5-*epi*-nakijiquinone U	**191**			
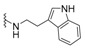	5-*epi*-nakijiquinone T	**192**			

**Table 5:** Meroterpenoids with a HQ fragment.

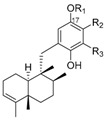					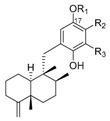				
**R_1_**	**R_2_**	**R_3_**	**Name**		**R_1_**	**R_2_**	**R_3_**	**Name**	
H	H	H	avarol	**196**	H	H	H	neoavarol	**197**
Ac	H	H	17-*O*-acetylavarol	**198**	Ac	H	H	17-*O*-acetylneoavarol	**199**
H	H	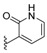	dysivillosin A	**200**	H	H	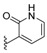	dysivillosin B	**201**
H	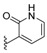	H	dysivillosin C	**202**	H	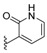	H	dysivillosin D	**203**

**Table 6:** Meroterpene-1,2-hydroquinones.

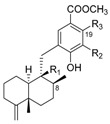					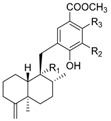				
**R_1_**	**R_2_**	**R_3_**	**Name**		**R_1_**	**R_2_**	**R_3_**	**Name**	
CH_3_	OH	OH	nakijinol C	**204**	CH_3_	OH	H	dictyoceratin A (smenospondiol)	**210**
CH_3_	OCH_3_	H	polyfibrospongol A	**205**	CH_3_	OCH_3_	OH	langconol A	**211**
CH_2_OH	OCH_3_	H	polyfibrospongol B	**206**	CHO	OCH_3_	H	langconol B	**212**
CHO	OCH_3_	OH	smenohaimien D	**207**	CHO	OCH_3_	OH	langconol C	**213**
CH_2_OH	OCH_3_	OH	19-hydroxy-polyfibrospongol B	**208**					
CH_2_OH (8-*epi*-)	OCH_3_	OH	smenohaimien E	**209**					

**Table 7:** Other meroterpenehydroquinones.

	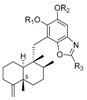				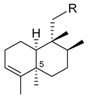		
**R_1_**	**R_2_**	**R_3_**	**Name**		**R**	**Name**	
H	H	H	nakijinol B	**214**	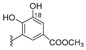	18-hydroxy-5-*epi*-hyrtiophenol	**219**
H	CH_3_	CH_3_	(−)-nakijinol E (+)-5-*epi*-nakijinol E	**215** **216**	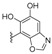 (5-*epi*-)	nakijinol A	**220**
H	CH_3_	H	smenohaimien C (nakijinol F)	**217**	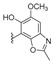	5-*epi*-nakijinol C	**221**
H	H	CH_3_	nakijinol G	**218**	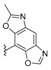	5-*epi*-nakijinol D	**222**
